# Automated 3D Tooth Segmentation and Dental Splint Synthesis from Intraoral Scans: A Deep Learning Pipeline for Computer-Aided Digital Dentistry

**DOI:** 10.3390/diagnostics16183039

**Published:** 2026-09-19

**Authors:** Berrin Çelik, Mehmet Özkaya, Mahmut Emin Çelik

**Affiliations:** 1 Department of Oral and Maxillofacial Radiology, Faculty of Dentistry, Ankara Yıldırım Beyazıt University, Ankara 06010, Türkiye; 2 Computer Engineering Department, Faculty of Engineering, Ankara University, Ankara 06830, Türkiye; 3 Electrical Electronics Engineering Department, Faculty of Engineering, Gazi University, Ankara 06570, Türkiye; 4 TUSAŞ-Kazan Vocational School, Gazi University, Ankara 06980, Türkiye

**Keywords:** 3D dental segmentation, computer-aided diagnosis, temporomandibular disorders, deep learning, dental splint design, mesh processing, intraoral scans

## Abstract

**Background/Objectives:** Temporomandibular disorders (TMDs) and sleep-related bruxism affect up to 40% and 31% of adults, respectively, and their management relies on accurate diagnostic assessment of the dental arch—including tooth identification, arch-form analysis, and occlusal-plane evaluation—followed by the fabrication of full-coverage hard occlusal stabilization splints. Current digital workflows still depend on manual CAD delineation for both diagnostic segmentation and splint design, introducing inter-operator variability. This study presents a computational framework that automates the diagnostic segmentation of 3D intraoral scans and the subsequent generation of patient-specific stabilization splints. **Methods:** Using the Teeth3DS+ dataset, 3D meshes are rendered into 32 views via a Fibonacci Hemisphere camera-placement strategy, and seven deep learning architectures are trained for pixel-level diagnostic classification of 34 dental structures. Predictions are back-projected onto the mesh through probability-aggregation voting and iterative graph diffusion; the occlusal plane is estimated by singular value decomposition of tooth centroids; and a volumetric dual-shell splint is synthesized by topological dilation and normal-based displacement. **Results:** UNet++ achieves the strongest performance (MPA 98.75%, mIoU 71.76%, Dice 75.20%), with its superiority over every competing architecture confirmed by both paired *t*-test and Wilcoxon signed-rank tests at the image and scan levels (all *p* < 0.001). On 60 held-out scans, back-projection and graph diffusion raise segmentation quality from a per-view mIoU of 62.0% to a mesh-level mIoU of 87.4% (Dice from 65.2% to 91.3%), with every held-out scan improving. Per-class analysis reveals near-ceiling accuracy on well-represented teeth; performance on the rarest and most posterior teeth (third molars) was markedly lower in preliminary experiments but improved substantially once the learning rate was selected via a validation-based sweep—a finding with direct implications for the diagnostic reliability of AI-assisted dental arch assessment and for the sensitivity of rare-class segmentation to training hyperparameters. **Conclusions:** The pipeline converts a raw intraoral scan into a diagnostically segmented model and a 3D-printable Michigan-type splint geometry without manual intervention, providing a foundation for accelerating the diagnostic-to-treatment cycle in TMD and bruxism management.

## 1. Introduction

The diagnostic assessment and clinical management of temporomandibular disorders (TMDs) and sleep-related bruxism represent one of the most pressing unresolved challenges in contemporary oral healthcare. Population-based studies estimate that signs or symptoms of TMD affect approximately 30 to 40 percent of adults, whereas the reported prevalence of awake and sleep bruxism ranges from roughly 8 to 31 percent depending on the diagnostic criteria and cohort examined [1,2]. When inadequately managed, these conditions contribute to masticatory muscle pain, accelerated and irreversible tooth wear, restorative and prosthetic failure, and a measurable reduction in oral-health-related quality of life [3,4]. Accurate diagnosis of these conditions requires a systematic assessment of the dental arch—including the identification and spatial classification of individual teeth, the evaluation of occlusal-plane orientation, and the detection of missing or malpositioned teeth—tasks that increasingly rely on three-dimensional (3D) imaging from intraoral scanners [5,6]. Full-coverage hard occlusal stabilization splints, of which the Michigan-type appliance is the canonical example, remain the most widely indicated reversible and non-invasive intervention for protecting the dentition, stabilizing the temporomandibular joint, and redistributing parafunctional occlusal loads [3,7]. Importantly, the stabilization splint also serves a diagnostic function: the patient’s clinical response to splint therapy—including changes in pain scores, muscle tenderness, joint loading, and condylar position—is a recognized diagnostic criterion that guides differential diagnosis and informs subsequent treatment decisions, including the need for further imaging, physiotherapy, or surgical referral [3]. Systematic reviews of randomized evidence further confirm that splint therapy yields measurable symptom reduction and structural protection of the dentition, reinforcing the central role of these devices in both the diagnostic workup and the ongoing management of orofacial parafunctional disorders [7].

The transition from analog to fully digital workflows has fundamentally reshaped both the diagnostic and fabrication pathways for occlusal appliances. Intraoral scanners now provide high-resolution, metrically accurate 3D surface meshes of the dentition, displacing conventional impressions and serving as the digital foundation for diagnostic assessment, orthodontic planning, crown and bridge restoration, implant guidance, and appliance manufacturing [6,7,8]. The expanding role of artificial intelligence, and of deep learning in particular, has further accelerated this transition, with convolutional neural networks now established across dental image diagnostics and analysis [6,9,10]. A critical prerequisite for any automated diagnostic or geometric workflow built on these scans is the accurate semantic segmentation of individual teeth, which delineates anatomical units and enables precise identification, classification, measurement, and downstream clinical reasoning—including the automated detection of missing teeth, supernumerary teeth, positional anomalies, and arch-form irregularities [11,12]. Despite these advances, the semantic segmentation of 3D intraoral meshes continues to pose significant technical obstacles—arising from the complex and variable topology of the dental arch, substantial inter-patient morphological diversity, the frequent presence of missing teeth or restorations, and the high computational cost of processing dense point clouds or meshes directly—leaving the diagnostic potential of fully automated 3D dental analysis largely unrealized.

Two paradigms currently coexist for splint production. In the conventional analog workflow, alginate or elastomeric impressions are poured into stone casts, mounted on a mechanical articulator, and the appliance is waxed and processed in acrylic resin by a technician—a labor-intensive sequence that introduces material- and operator-dependent variability at every stage. The digital workflow replaces these steps with intraoral scanning, computer-aided design (CAD), and computer-aided manufacturing (CAM) through milling or additive 3D printing, reducing chairside and adjustment time while improving reproducibility [13,14]. However, even state-of-the-art digital pipelines still require an operator to manually delineate dental structures, define the appliance outline, and sculpt the occlusal morphology within commercial CAD environments such as 3Shape or exocad [13,15]. This manual design stage is the principal bottleneck of the digital workflow: it is time-consuming, demands specialized expertise, and remains a documented source of inter-operator variability, with the trueness of the resulting devices shown to differ according to the operator and the design software employed [14,15]. Recent attempts to automate splint design with artificial intelligence have been benchmarked against human technicians but still operate within, rather than replace, the manual CAD paradigm [15].

Deep learning for 3D shape understanding has matured into several methodological families that are directly relevant to dental segmentation. Point-based networks such as PointNet [16] and PointNet++ [17] learn permutation-invariant features directly from unordered point sets, while graph- and edge-convolution methods including the Dynamic Graph CNN (DGCNN) [18] explicitly model local geometric neighborhoods. Building on these foundations, mesh- and point-cloud-specific architectures have defined the state of the art in tooth segmentation: MeshSegNet introduced multi-scale geometric feature learning on raw dental surfaces [11], TSegNet employed a centroid-guided two-stage design for efficiency and accuracy [12], two-stream graph convolutional networks exploited the complementary cues of coordinates and surface normals to refine inter-tooth boundaries [19], and more recent formulations such as the two-stage iMeshSegNet [20] and the graph-neural TeethGNN [21] report tooth-level accuracies in the high-ninety-percent range. The multi-stage TSegLab pipeline further reports teeth-segmentation and identification rates above 97 percent on the Teeth3DS benchmark [22,23]. Despite their accuracy, these methods generally demand substantial memory and bespoke architectural engineering to operate on dense geometric primitives, and—crucially—they terminate at the labeling stage without producing a manufacturable device [11,12,19,20,21,22].

An alternative and computationally efficient strategy is to project 3D geometry into multiple 2D views and to leverage mature image-based networks. This multi-view paradigm was popularized by the Multi-View Convolutional Neural Network (MVCNN) for 3D shape recognition [24] and subsequently extended by view-pooling and relation-modeling approaches such as RotationNet [25] and View-GCN [26], which demonstrated that aggregating evidence across rendered viewpoints can match or exceed direct 3D processing while benefiting from large pretrained 2D backbones. Within the 2D domain, encoder–decoder segmentation architectures such as U-Net [27] and the atrous-convolution-based DeepLabV3+ [28], typically built on deep residual backbones such as ResNet [29], provide well-understood, high-capacity feature extractors. Adapting the multi-view strategy to intraoral meshes is therefore attractive: it sidesteps the memory cost of dense 3D operators and exploits transferable 2D representations, but it introduces the complementary challenges of viewpoint coverage, occlusion, and the consistent back-projection of 2D predictions onto the original 3D surface.

**Figure 1:**
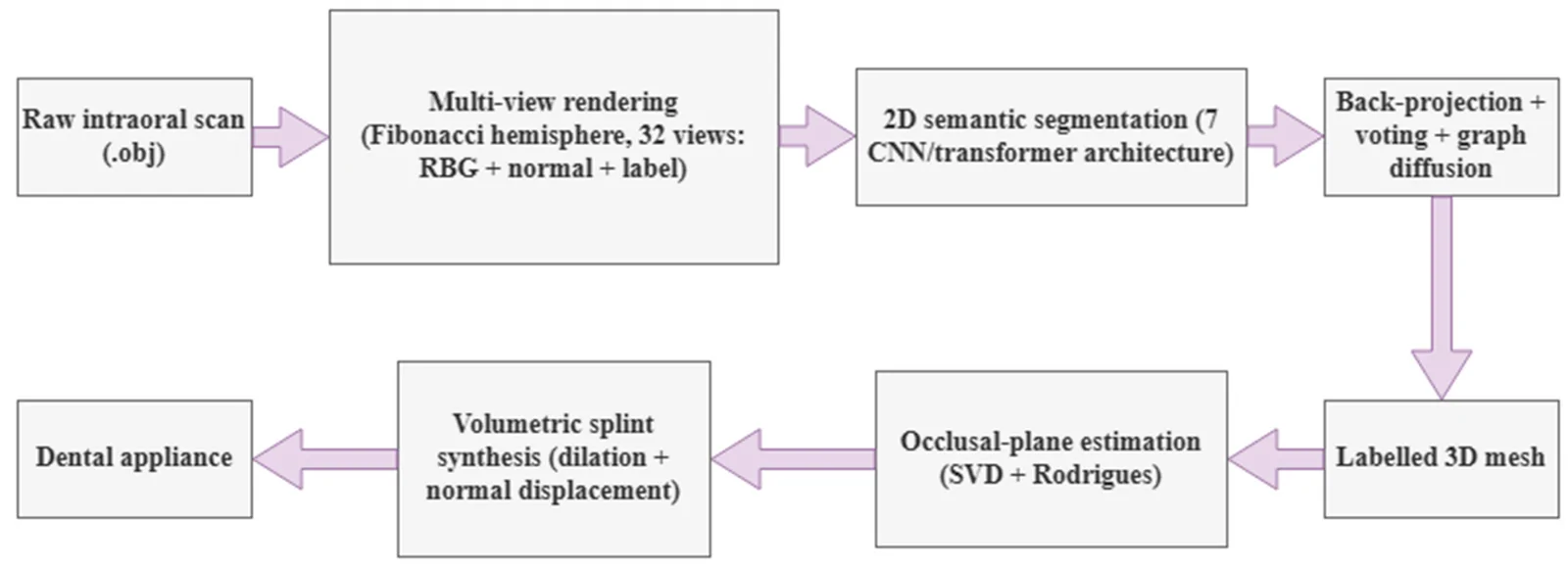
Overview of the proposed end-to-end pipeline: from a raw intraoral scan, through multi-view rendering and 2D semantic segmentation, 3D back-projection, occlusal-plane estimation, and volumetric splint synthesis, to a 3D-printable stabilization splint.

Taken together, the literature reveals a clear and persistent gap: a rich body of work performs accurate tooth segmentation but stops at the labeled surface [11,12,19,20,21,22], whereas digital splint fabrication continues to rely on operator-driven CAD [13,14,15], and no existing study, to our knowledge, bridges the two with a fully automated end-to-end pipeline that converts a raw intraoral scan into a 3D-printable stabilization splint—through diagnostic segmentation, robust 3D reconstruction, automated occlusal-plane estimation, and volumetric appliance synthesis—using a multi-view deep learning approach and without any manual CAD intervention. To close this gap, we propose an integrated computational framework, illustrated in Figure 1, that treats diagnostic segmentation not as the final objective but as the enabling intermediate stage of a complete diagnostic-to-intervention workflow. Individually, the framework’s stages build on established techniques—multi-view rendering and back-projection, singular-value-decomposition-based plane fitting, and mesh dilation/offsetting are each well-known operations in their respective domains. The contribution of this work is not any single stage in isolation, but their integration into a single, fully automated pipeline that has not, to our knowledge, been previously assembled end-to-end for this task. We summarize this integration in four components.

First, we introduce a multi-view back-projection segmentation framework that renders each intraoral mesh into 32 complementary 2D views using an optimized Fibonacci-hemisphere camera-placement strategy, enabling seven state-of-the-art 2D segmentation networks to be applied to the diagnostic classification of 3D dental structures. Second, we develop a probability-aggregation voting scheme coupled with an iterative graph-diffusion algorithm that back-projects 2D predictions onto the mesh and resolves occlusion-induced holes, yielding a continuous, denoised, and manifold labeled surface. Third, we formulate an automated occlusal-plane estimation and alignment procedure based on singular value decomposition of tooth centroids and Rodrigues’ rotation—providing a quantitative, operator-independent diagnostic measurement that is a prerequisite for both TMD assessment and prosthetic planning—followed by a volumetric splint-synthesis stage that combines topological dilation for gingival extension with normal-based displacement for uniform clinical thickness. Fourth, we integrate these components into a single, reproducible end-to-end pipeline that transforms a raw intraoral scan into a diagnostically segmented model and subsequently into a clinically meaningful, 3D-printable Michigan-type stabilization splint without manual CAD modeling, establishing a scalable foundation for next-generation computer-aided diagnostic and therapeutic systems in digital dentistry. To the best of our knowledge, this is therefore the first framework to unify multi-view diagnostic tooth segmentation, automated occlusal-plane estimation, and volumetric splint synthesis within a single automated workflow.

## 2. Materials and Methods

### 2.1. Dataset and Data Preprocessing

#### 2.1.1. Dataset

In this research, we utilize the Teeth3DS+ dataset [23], which contains high-fidelity upper and lower oral scans and serves as a benchmark collection for intraoral mesh analysis. The dataset is organized into seven distinct partitions containing upper and lower oral scans. For the scope of this study, the primary partition was employed, comprising 399 3D object models. To facilitate the training and evaluation of deep learning architectures, the data was partitioned into a training set of 300 models and a test set of 99 models. Each oral scan is associated with a unique identifier and consists of an object file (.obj) for the 3D geometry and a metadata file (.json) containing patient IDs, jaw types, FDI tooth labels, and instance information. The overall dataset composition and the multi-view rendering configuration are summarized in Table 1.

Of the 399 scans processed in this study, 300 constituted the training/validation pool and 99 formed a held-out test set, rendered independently and never accessed during training or model selection. Within the 300-scan pool, the 9600 rendered views were split at the individual-view level into 7680 training and 1920 validation views (80/20, random, seed = 42); the 99-scan test set (3168 views) was reserved exclusively for the final evaluation reported in Section 3, and was not used for hyperparameter tuning, learning-rate scheduling, or model checkpoint selection (see Section 2.3).

Table 1 summarizes the dataset composition; the underlying class distribution is reported in full in Supplementary Table S1 (Supplementary Material). Background and gingiva together account for 87.5% of all labeled pixels across the corpus, consistent with these two classes occupying the largest contiguous surface area of each rendered view. Among the 32 tooth classes, representation varies with anatomical position: the first molars (16, 26, 36, 46) are the most represented (0.70–0.84% of pixels each), while the third molars (18, 28, 38, 48) are the least represented: each individual third-molar class accounts for only 0.03–0.06% of pixels (Table S1), roughly 20-fold less than a typical first-molar class (0.70–0.84%), and the four third-molar classes together account for 0.155% of all labeled pixels. This distribution reflects normal anatomical prevalence and viewpoint coverage (Section 2.1.2) rather than a sampling artifact, and it is referenced again in Section 3.3 when interpreting per-class segmentation performance.

#### 2.1.2. Multi-View Rendering (3D Models into 2D Views)

**Figure 2:**
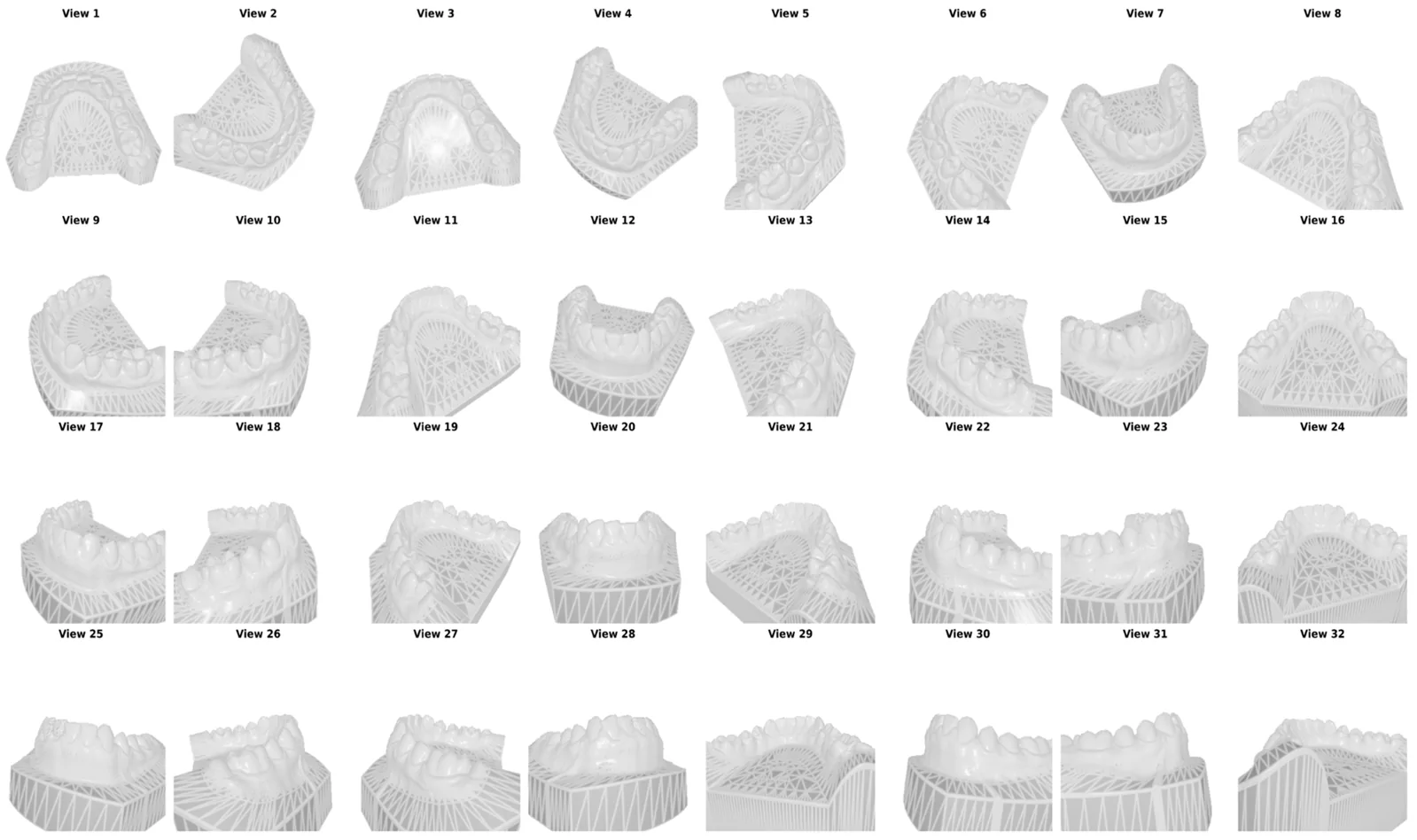
Views taken from a single 3D model.

By employing the PyTorch3D (v0.7.8) library and a customized Fibonacci Hemisphere formula for camera placement, we generate 32 distinct 2D views for each 3D model. These views are then used to train and compare deep learning models. Given the computational complexity of direct 3D mesh processing, a multi-view projection strategy was adopted to convert 3D oral scans into 2D representations. To ensure optimal coverage of the dental structures, a camera placement strategy based on the Fibonacci Sphere algorithm was implemented and subsequently modified into a Fibonacci Hemisphere. The camera positions were calculated according to the optimized equation in (2), in which a variable y_min = 0.5 restricts the sampling to the superior portion of the model where the teeth are visible. Example views taken from a single 3D model are given in Figure 2.

(1) $$p_{i}=\left[x_{i}y_{i}z_{i}\;\right]=R\times \left[\sqrt{1-y_{i}^{2}}\times cos(\varphi i)\;1-\;\frac{2i}{n-1}\;\sqrt{1-y_{i}^{2}}\times sin(\varphi i)\;\right]$$

(2) $$p_{i}=\left[x_{i}y_{i}z_{i}\;\right]=R\times \left[\sqrt{1-y_{i}^{2}}\times cos(\varphi i)\;1-\;\frac{i}{n-1}\times (1-y_{min})\;\sqrt{1-y_{i}^{2}}\times sin(\varphi i)\;\right]$$

where *p_i_* is the position of the *i*-th point, *φ* = π × (3 − √5) is the golden angle, and *R* is the radius of the sphere.

The view count of 32 was selected to balance mesh-coverage completeness against computational cost: preliminary coverage analysis indicated that additional views beyond this range yield diminishing marginal increases in surface coverage due to the geometric redundancy inherent to a hemispherical camera arrangement, while substantially increasing rendering time, dataset size, and per-scan inference latency. Residual occluded regions (e.g., interproximal surfaces) are recovered by the graph-diffusion step (Section 2.5) rather than by increasing view density.

#### 2.1.3. Normal and Label Maps

In addition to RGB images, the preprocessing pipeline generated two critical supplementary data types for each view:

- Normal Maps: These maps store surface normal vector information for each pixel, which is essential for the subsequent 3D reconstruction and back-projection phase. An example normal map can be seen in Supplementary Figure S1.
- Label Maps: Utilizing the FDI labels from the JSON metadata, each pixel in the rendered images was assigned a specific class ID. A global light source was applied during the rendering process to ensure color consistency and prevent lighting artifacts from affecting the pixel-level classification. An example label map can be seen in Supplementary Figure S1.

### 2.2. Model Architectures and Training

The semantic segmentation task is implemented using the PyTorch (v2.5.1) deep learning framework. To identify the most suitable network for dental mesh classification, seven state-of-the-art encoder–decoder architectures were developed and evaluated concurrently under identical conditions. UNet provides the canonical symmetric encoder–decoder with skip connections that recover spatial resolution for precise boundary localization. DeepLabV3 [30] employs atrous convolutions to enlarge the receptive field without loss of resolution, while DeepLabV3+ extends it with a ResNet50 backbone and Atrous Spatial Pyramid Pooling (ASPP) to capture multi-scale context and refine boundaries. MANet [31] augments the decoder with multi-scale attention to emphasize salient dental regions. UNet++ [32] redesigns the skip pathways as densely nested, convolutionally bridged connections that reduce the semantic gap between encoder and decoder. The transformer-based models capture long-range dependencies through self-attention: SegFormer [33] couples a hierarchical transformer encoder with a lightweight all-MLP decoder, and TransUNet [34] combines a convolutional backbone with a transformer bottleneck within a UNet-style decoder. All seven models were trained with the dataset, preprocessing, optimizer, and training schedule held constant, so that any performance difference is attributable to architecture alone; the full comparative evaluation from which the best-performing model was selected is reported in Section 3.

All seven architectures used encoders initialized from ImageNet-pretrained weights (ResNet34 for UNet, DeepLabV3 and MANet; EfficientNet-B4 for UNet++; ResNet50 for DeepLabV3+; MiT-B3 for SegFormer; ResNet50 for TransUNet’s convolutional branch), so training in every case corresponds to fine-tuning rather than learning from random initialization. Because pretrained initialization substantially accelerates convergence relative to training from scratch, a fixed 10-epoch fine-tuning budget is a common and reasonable choice for this class of models, though it was not independently tuned per architecture. All seven models were trained under an identical schedule (optimizer, learning rate, batch size, and epoch count) to isolate architectural differences as the primary source of variation; we note this as a deliberate design choice for a controlled comparison, and, as a limitation, one that does not rule out the possibility that individual architectures—particularly the transformer-based ones—could reach higher performance under an architecture-specific schedule.

**Figure 3:**
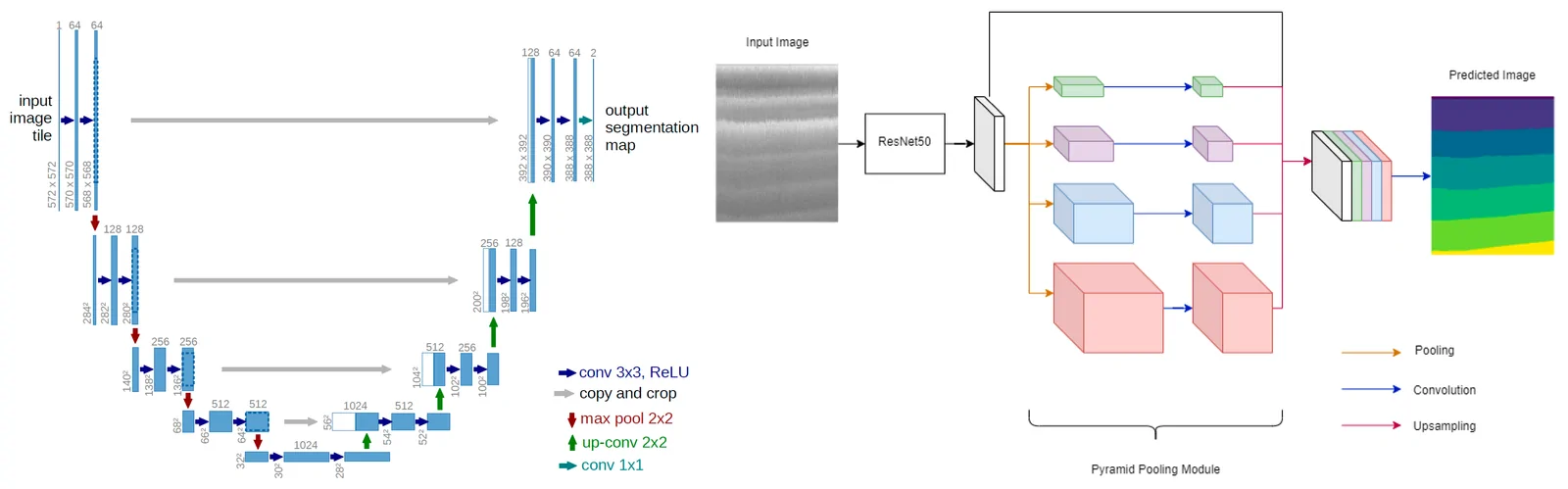
UNet architecture (**left**) and DeepLabV3+ (**right**).

The two representative encoder–decoder designs are illustrated in Figure 3.

### 2.3. DataLoader and Training Setup

To facilitate efficient model training, a custom PyTorch (v2.5.1) DataLoader class was developed. This class performs critical preprocessing tasks, including the conversion of rendered images from BGR to RGB format. To ensure accurate pixel-level classification, the loader utilizes a predefined dictionary to map RGB color values to their corresponding FDI class IDs. The colour-to-class mapping applied by the loader is illustrated by the rendered label maps in Supplementary Figure S1.

A significant challenge in dental image segmentation is ensuring that every pixel precisely matches a known class. To mitigate potential color discrepancies arising from rendering artifacts, a Squared Euclidean Distance formula was implemented:

(3) $$d^{2}={\left(R_{p}\;-\;R_{c}\right)}^{2}+\;{\left(G_{p}\;-\;G_{c}\right)}^{2}+\;{\left(B_{p}\;-\;B_{c}\right)}^{2}$$

This formula calculates the distance between the unique pixel colors in the image and the predefined palette colors, assigning each pixel to the class with the minimum distance. Model selection during training relied exclusively on a held-out validation split (Section 2.1.1): a ReduceLROnPlateau scheduler (factor 0.5, patience 3 epochs) reduced the learning rate based on validation mIoU, and the checkpoint with the highest validation mIoU was retained as the final model for each architecture. The held-out test set was accessed exactly once per architecture, after training was complete, and was never used for hyperparameter tuning or model selection. The base learning rate was additionally verified with a validation-only sweep: five configurations (learning rate ∈ {5 × 10^−5^, 1 × 10^−4^, 2 × 10^−4^}, batch size ∈ {2, 4, 8}) were trained under an abbreviated protocol on UNet++, and the two most competitive learning rates were extended to 8 epochs on the same validation subset. Learning rate 2 × 10^−4^ consistently outperformed 1 × 10^−4^ at every checkpoint from 3 to 8 epochs (validation mIoU 0.14 vs. 0.10 at epoch 3; 0.68 vs. 0.41 at epoch 8) and was adopted for all seven architectures in the full 10-epoch training reported in this paper (Table 2). The full sweep is reported in Supplementary Table S3. Batch size and epoch count were not swept and were fixed a priori to common defaults for fine-tuning ImageNet-pretrained encoders on a dataset of this size. To mitigate the pronounced class imbalance quantified in Table S1, an equally weighted combination of Dice loss and Focal loss (0.5 × Dice + 0.5 × Focal) was used as the training objective in place of plain cross-entropy, following common practice for imbalanced segmentation tasks. The training hyperparameters are given in Table 2.

### 2.4. Predictions and Evaluation

To evaluate the efficacy of the proposed architectures, identical preprocessing operations were executed on the held-out test set of 99 distinct 3D models. Upon completion of the training phase, these unseen models were used as input to generate semantic segmentation masks for each of the 32 rendered views. Model performance was evaluated using Mean Pixel Accuracy (MPA), mean Intersection over Union (mIoU), and the Dice similarity coefficient (DSC), each reported as a mean with its standard deviation across all evaluation images.

In addition to the per-view (2D) metrics above, we quantified the fidelity of the final 3D reconstruction by computing mesh-level accuracy, IoU, and Dice directly on the back-projected and graph-diffused mesh, comparing the predicted per-face label (Section 2.5) against the original per-vertex Teeth3DS+ annotation of the same mesh, reduced to a per-face label by majority vote over each face’s three vertices. Because the 2D training split of Section 2.1.1 was made at the view level, every scan in the 300-scan pool contributed views to training, and the original meshes of the 99-scan test set were not retained after rendering. A genuine held-out mesh-level evaluation therefore required a separate, scan-level protocol: the 300-scan pool was partitioned into 240 training scans and 60 held-out scans (stratified by jaw and by third-molar presence; seed 42), an auxiliary UNet++ was trained on views of the 240 training scans only, under the identical protocol of Section 2.3 (validation split drawn within those 240 scans; best validation mIoU 0.774 versus 0.781 for the deployed model), and both per-view 2D metrics and mesh-level metrics were then computed on the 60 held-out scans, which this auxiliary model had never seen in any view. Because both quantities are measured on the same unseen scans with the same model, their difference isolates the contribution of the back-projection and graph-diffusion stages. The per-view figures from this auxiliary protocol are reported only as the matched 2D baseline for that comparison; the held-out test-set results of Table 3 remain the reference 2D evaluation.

### 2.5. 3D Reconstruction of the Segmented Object

Once the 2D segmentation predictions were obtained from all 32 rendered views, the pixel-level class labels were mapped back onto the original 3D mesh surface through a back-projection procedure. Because a single mesh face may be visible in several views—each potentially yielding a different prediction—the framework aggregates the full 34-class probability vectors across all views in which a given face appears and assigns the final label by selecting the class with the highest cumulative confidence. Mesh faces that were not captured in any rendered view due to geometric occlusion (e.g., interproximal surfaces and deep fossae) are resolved through an iterative graph-diffusion algorithm that propagates label information from classified neighbors until every face on the mesh is assigned a class. The result is a continuous, denoised, and manifold-consistent labeled surface suitable for downstream geometric processing (Supplementary Figure S2). The diffusion is applied iteratively, advancing one face ring per pass, until every face carries a label or a cap of 20 iterations is reached.

### 2.6. Occlusal Plane Estimation

Accurate identification of the occlusal plane is a prerequisite for the subsequent splint design. The geometric centroid of each segmented tooth was first computed from its constituent mesh vertices, reducing the complex 3D arch geometry to a representative set of landmark points. A best-fit plane was then derived from these centroids using Singular Value Decomposition (SVD), which identifies the plane of least variance—effectively the mathematical equivalent of the clinical occlusal plane. Because SVD introduces a 180-degree directional ambiguity, the plane normal was oriented toward the occlusal surface by referencing the spatial relationship between the gingival centroid and the tooth centroids. Finally, the entire mesh was repositioned so that the estimated occlusal plane aligns horizontally with the global coordinate frame, using a rotation derived from Rodrigues’ formula (Supplementary Figure S3).

### 2.7. Automated Splint Synthesis and Volumetric Modeling

The splint geometry was synthesized directly from the segmented and aligned mesh through a three-stage geometric pipeline. First, the dental region of interest was isolated by masking all non-dental structures (background and gingiva), retaining only the tooth surfaces as the initial seed geometry. To improve clinical retention, this seed region was then expanded beyond the cervical line onto the marginal gingival tissue through an iterative topological dilation process, extending coverage by approximately 2.5 mm—a parameter that can be adjusted to produce either a tooth-borne or gingiva-extended design depending on the clinical indication. The resulting surface was extracted as an independent sub-mesh (the inner shell), and a uniform outer shell was generated by displacing each vertex along its local surface normal by a scalar offset of approximately 2.5 mm, producing a dual-shell volumetric representation whose wall thickness approximates the target offset across most of the arch, with local deviations at high-curvature regions quantified below (Supplementary Figure S4). The pipeline thus converts the segmented scan into a full-coverage stabilization-splint geometry without manual CAD intervention. The gingival extension reported here corresponds to three dilation iterations, and the outer shell is displaced along area-weighted vertex normals.

The cervical line is defined operationally as the boundary between the segmented tooth classes and the gingiva class in the aligned mesh, providing a reproducible, segmentation-driven reference for gingival extension without requiring a separate curvature-based detector. We validated the resulting dual-shell geometry on a representative case: the shell was watertight across 5 of 6 connected components, with the boundary-edge construction confirmed to be fully edge-manifold throughout; the one exception was traced to a small cluster of non-manifold vertices at a point of natural inter-tooth contact, a localized and identifiable case for targeted mesh repair. Wall thickness, measured as the nearest-surface distance between the outer and inner shells, averaged 2.08 mm (SD 0.57 mm) against the 2.5 mm target, with the expected concentration of deviation at interproximal embrasures and cusp tips (Section 3.5). We further quantified manufacturability via a single-axis draft-angle analysis referenced to the estimated occlusal-plane normal, which identified 36.8% of the outer-shell surface as locally undercut relative to a rigid vertical insertion path—a standard prosthodontic screening heuristic that informs where an undercut block-out step (Section 4.6) would have the greatest effect for a rigid, hard-acrylic appliance.

During the preparation of this manuscript, the authors used GPT-5.6 Luna for English-language editing, including grammar, spelling, punctuation, and readability improvements. The authors reviewed and edited all output and take full responsibility for the content of this publication.

## 3. Results

### 3.1. Segmentation Performance and Benchmarking

The proposed end-to-end pipeline was evaluated along three complementary axes: the accuracy of the multi-view semantic segmentation, the geometric integrity of the 3D back-projection and reconstruction, and the structural quality of the synthesized splint. This section reports each in turn. All segmentation metrics were computed on the held-out test set of 99 intraoral scans, which was processed with the identical preprocessing pipeline used during training and was not seen by the networks at any stage.

To provide a rigorous and clinically meaningful evaluation, all models were assessed using three complementary pixel-level metrics computed per rendered image and then aggregated over the full test set: Mean Pixel Accuracy (MPA), mean Intersection-over-Union (mIoU), and the Dice similarity coefficient (DSC). The Dice coefficient was included because it is the de facto standard overlap metric in medical image segmentation and is more sensitive to small, under-represented structures than raw pixel accuracy. For every metric we report the mean and its standard deviation across all evaluation images, and inter-model differences are further quantified through paired significance testing (Section 3.2).

As summarized in Table 3, MPA remains tightly clustered across all architectures (98.12–98.75%; a spread of only 0.63 percentage points), confirming that pixel-level accuracy is saturated and dominated by the abundant background and gingival regions that occupy the majority of each rendered view. Consequently, MPA is a weak discriminator between competing models and is most meaningfully interpreted alongside the boundary-sensitive overlap metrics.

The mIoU and Dice metrics are considerably more discriminative, spanning 66.71–71.76% and 70.93–75.20% respectively. UNet++ attains the highest overlap scores (mIoU 71.76%, Dice 75.20%); TransUNet, DeepLabV3+, and SegFormer form a statistically indistinguishable middle cluster (70.12–70.29% mIoU; Table 4), followed by UNet and DeepLabV3 (68.27–68.49% mIoU), while MANet records the lowest region overlap (66.71%/70.93%). The ≈5.1-percentage-point mIoU gap between the strongest and weakest models, set against their near-identical MPA values, demonstrates that architectural differences reside primarily in the ability to delineate fine inter-tooth boundaries and to recover rare classes rather than in gross classification accuracy. The advantage of the nested-skip UNet++, and the broadly comparable performance of the three transformer-influenced middle-cluster architectures over the plain CNN baselines (UNet, DeepLabV3, MANet), indicates that dense multi-scale aggregation and self-attention both help resolve the narrow interproximal regions where adjacent teeth meet, identifying these decoder families as promising directions for further improving boundary fidelity. The large standard deviations (≈15 percentage points for mIoU) reflect strong per-image variability driven by the rare, poorly covered distal molars, motivating the per-class analysis in Section 3.3.

**Figure 4:**
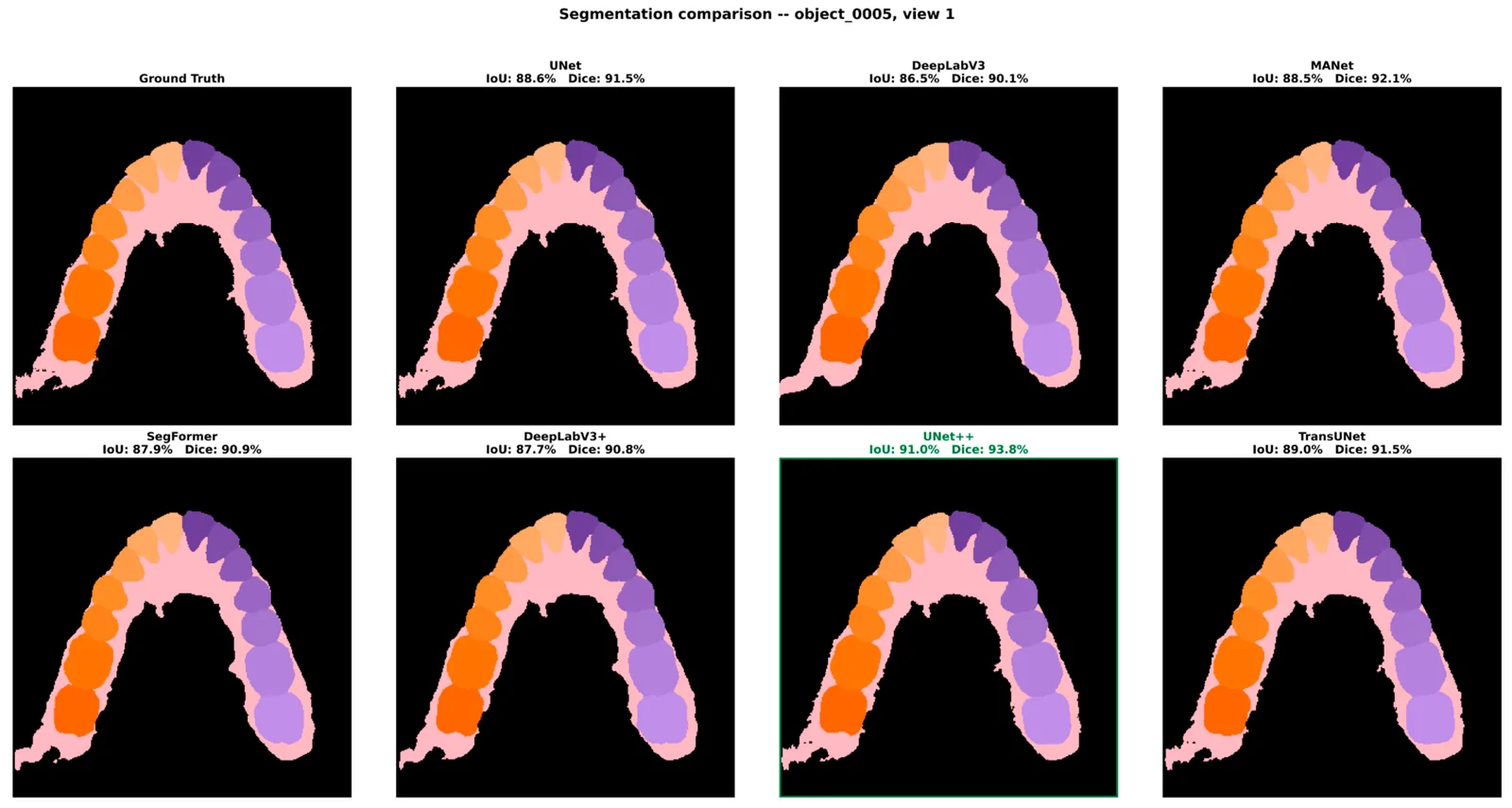
Qualitative comparison of the seven architectures on a representative held-out view (object_0005), shown against the ground-truth labels; the best-performing architecture (UNet++) is outlined.

**Figure 5:**
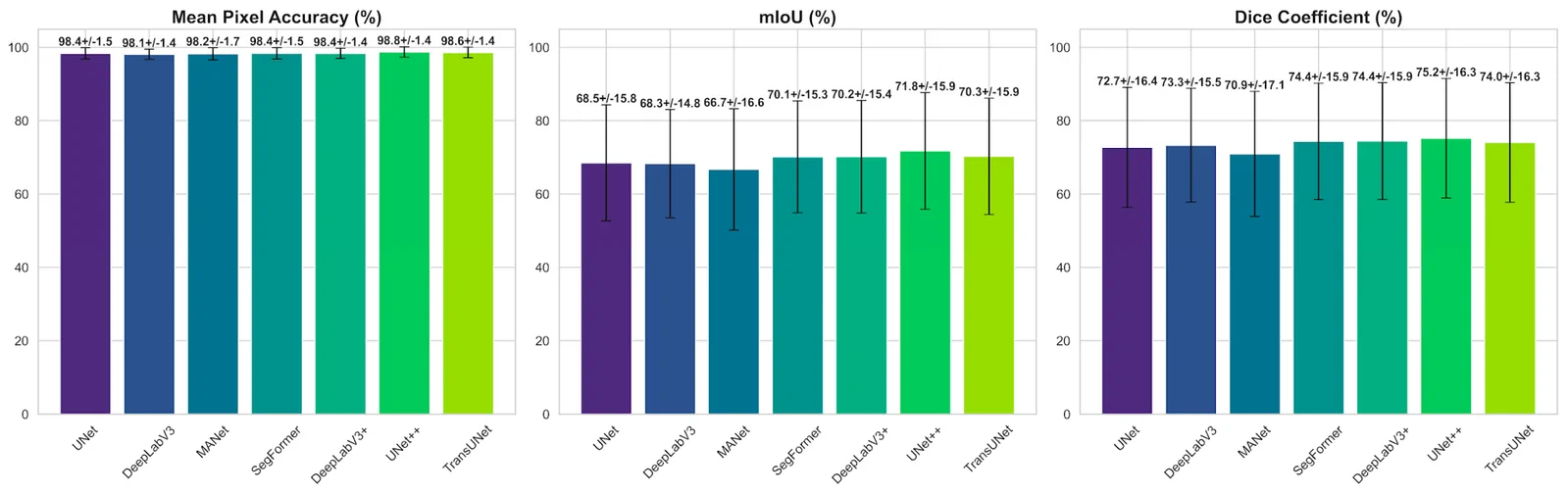
Mean segmentation performance with standard-deviation error bars for MPA, mIoU, and Dice across the seven architectures. The near-flat MPA panel contrasts with the clearly tiered mIoU and Dice panels, in which UNet++ leads and the three mid-cluster architectures follow.

Figure 4 shows a qualitative comparison of the seven architectures on representative held-out views, while Figure 5 presents the aggregate results as per-model bar charts of MPA, mIoU, and Dice with standard-deviation error bars. The MPA bars are virtually indistinguishable in height across all seven models, providing immediate visual confirmation that pixel-level accuracy is saturated, whereas the mIoU and Dice bars display a clearly tiered structure in which UNet++ forms the highest bar, followed by the three statistically indistinguishable mid-cluster architectures (TransUNet, DeepLabV3+ and SegFormer), with MANet the shortest. The contrast between the near-uniform MPA panel and the tiered overlap panels substantiates the conclusion that mIoU and Dice are the informative criteria for model selection in this fine-grained, 34-class task, and that the choice of architecture materially affects boundary-level performance even when overall pixel accuracy appears comparable.

### 3.2. Statistical Significance of Model Differences

**Figure 6:**
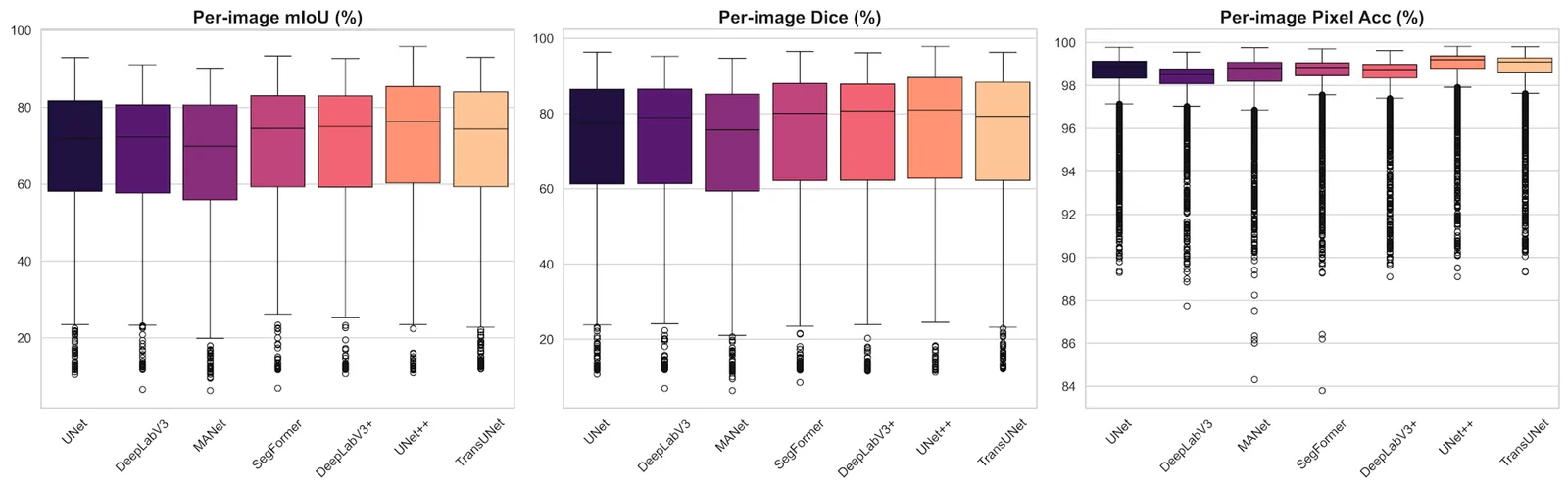
Per-image distributions of mIoU, Dice, and pixel accuracy across the test views. The left-skewed, heavy-tailed shape—with numerous low-outlier images—motivates the use of non-parametric significance testing.

Because the reported metrics are means over a large sample of evaluation images, we tested whether the differences between architectures are statistically significant rather than incidental. For every model pair we computed paired comparisons on the per-image mIoU distributions using both a paired Student’s *t*-test and the non-parametric Wilcoxon signed-rank test; the latter was included because the per-image metric distributions are left-skewed and heavy-tailed (Figure 6).

Across the 21 pairwise comparisons, the majority of model differences were statistically significant under both tests (*p* < 0.05), confirming that the ranking in Table 3 is largely robust and not attributable to sampling noise. The superiority of UNet++ over every competing architecture was significant under both tests (all *p* < 0.001). Three exceptions emerged among the mid-ranked architectures—SegFormer vs. DeepLabV3+, SegFormer vs. TransUNet, and DeepLabV3+ vs. TransUNet—none of which reached significance under the paired *t*-test, indicating that these three architectures are statistically indistinguishable from one another in mean per-image mIoU (Table 4).

Because the 32 rendered views of a given scan are not independent observations—they share patient-level anatomy, scan quality, and class composition—the significance testing was repeated after aggregating per-image scores to one mean value per scan (n = 99), rather than treating all 3168 views as independent samples. Of the 21 pairwise comparisons, 20 retained the same significance conclusion at the scan level. The exception was UNet vs. DeepLabV3 (Δ mIoU = 0.22 percentage points), which was significant at the image level (paired *t*-test *p* = 0.046) but not at the scan level (*p* = 0.186; Wilcoxon *p* = 0.054), indicating that the image-level test had overstated the significance of this particular, near-zero difference due to the non-independence of views within a scan. All other pairwise conclusions—including the superiority of UNet++ over every other architecture and the mutual indistinguishability of the SegFormer/DeepLabV3+/TransUNet middle cluster—were robust to this correction.

### 3.3. Per-Class and Anatomical Performance Analysis

Examination of the per-class behavior underlying the averaged metrics reveals a consistent anatomical pattern. The large, morphologically distinctive structures—gingiva, background, and the central and lateral incisors—were segmented most reliably, owing to their characteristic silhouettes and abundant pixel representation across the 32 views. Performance was lowest for the premolars and for the most posterior molars: premolars are frequently confused with their immediate neighbors because of their close morphological similarity and the gradual transition in crown shape along the arch, whereas the distal molars suffer from limited and oblique viewpoint coverage and partial occlusion by adjacent structures, which reduces the number of views in which they are fully visible. Boundary regions between adjacent teeth accounted for the majority of residual misclassifications, consistent with the gap observed between the near-perfect MPA and the more moderate mIoU. These observations identify class imbalance and inter-tooth boundary ambiguity—rather than gross misclassification—as the dominant error modes of the framework.

**Figure 7:**
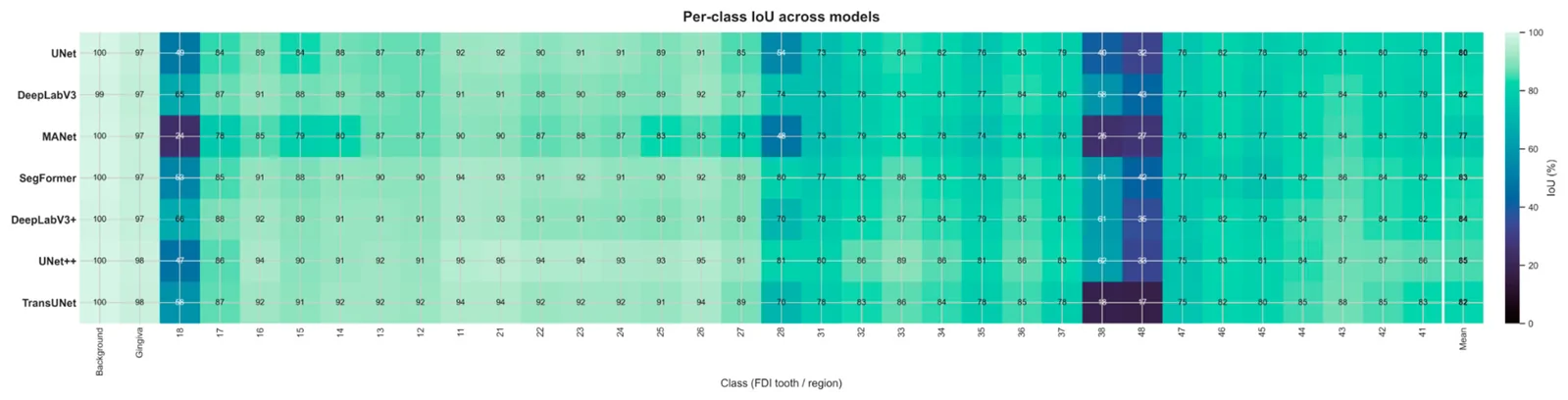
Per-class IoU across all 34 present classes for the seven architectures. Near-ceiling values on gingiva, background, and mid-arch teeth contrast with the third-molar rows (FDI 18/28/38/48), where performance is lowest across all architectures but no longer collapses to zero, following the learning-rate correction described in Section 2.3.

These qualitative observations are quantified in the per-class IoU heatmap (Figure 7). Every architecture segments the dominant structures almost perfectly (Background IoU ≈ 0.99–1.00; Gingiva ≈ 0.97–0.98) and recovers the anterior and mid-arch teeth reliably (IoU ≈ 0.73–0.95). Performance is consistently lowest on the terminal molars: the third molars (FDI 18, 28, 38, 48) remain the hardest classes for every model, with IoU ranging from 0.17 (TransUNet, tooth 48) to 0.81 (UNet++, tooth 28)—a wide but no longer collapsed range, in marked contrast to the complete failures (IoU = 0.00) observed under the original lr = 1 × 10^−4^ protocol before the validated learning-rate correction described in Section 2.3. TransUNet is now the weakest architecture specifically on teeth 38 and 48 (IoU 0.17–0.18), while the upper third molars are recovered most reliably by DeepLabV3+ and UNet++ (IoU 0.66 on tooth 18 and 0.81 on tooth 28, respectively); tooth 48 remains the hardest class for every architecture (IoU 0.17–0.43). On these classes, both precision and recall are moderate rather than near-zero (e.g., TransUNet on tooth 38: precision 0.71, recall 0.19; MANet on tooth 18: precision 0.55, recall 0.30), indicating that the residual error is now predominantly one of imprecise boundary localization on a partially recognized structure rather than outright non-detection. This is consistent with the extreme class imbalance among the third molars, which occupy as little as 0.03% of the labeled pixels and are frequently oblique or partially occluded across the 32 views. The averaged mIoU therefore still reflects a skewed per-class distribution—near-ceiling performance on well-represented teeth and comparatively weaker, but no longer near-zero, performance on the rarest distal molars—with class imbalance remaining the principal factor depressing the aggregate mIoU below its pixel-accuracy ceiling, and with the residual gap now substantially narrower than in the pre-correction protocol (Section 2.3).

### 3.4. 3D Back-Projection and Reconstruction Quality

**Figure 8:**
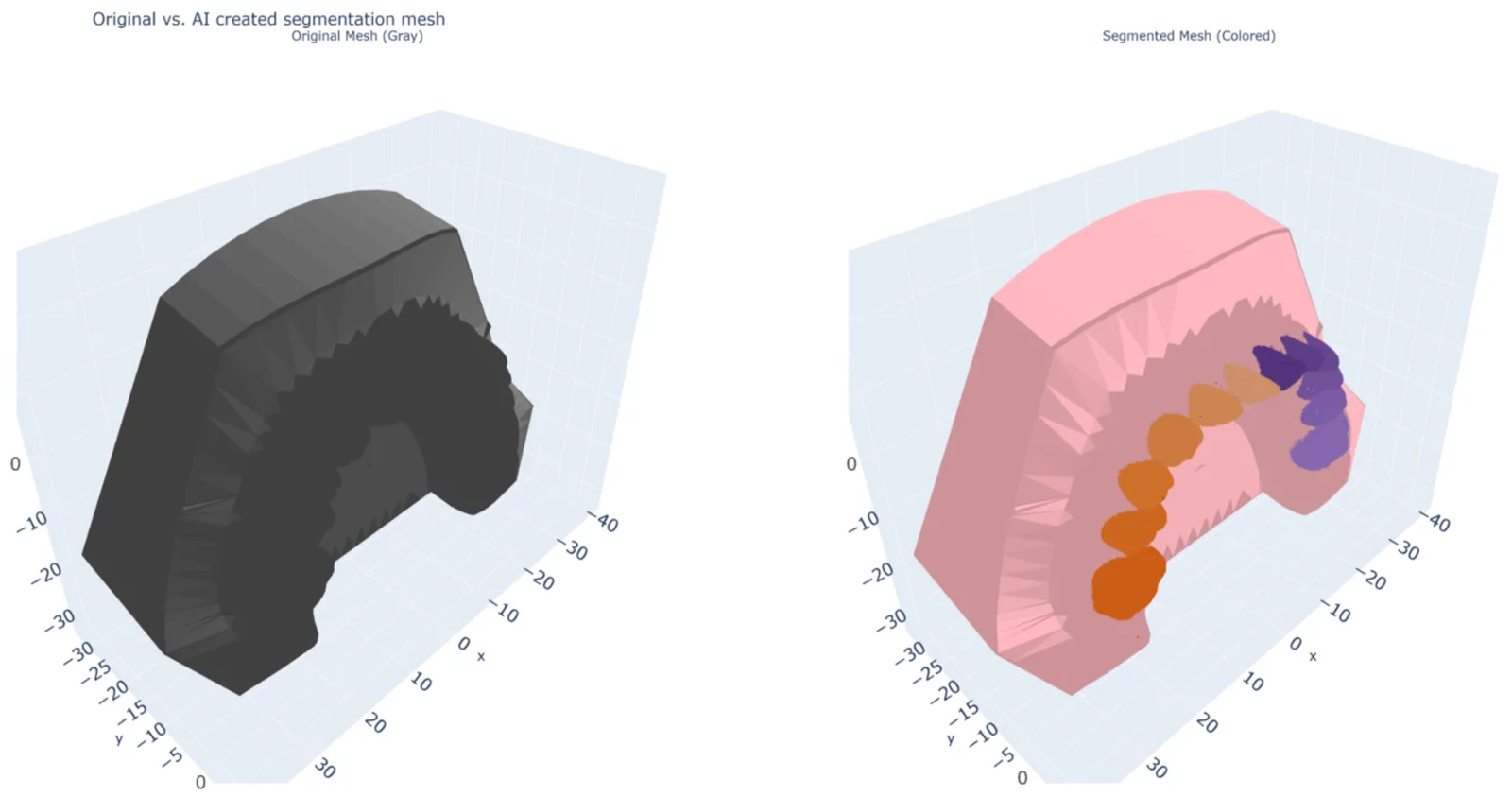
Original mesh (gray) and the corresponding AI-generated segmentation (colored), shown for a representative scan.

The efficacy of the 3D back-projection mechanism, comprising probability-aggregation voting and the Iterative Graph Diffusion algorithm, was confirmed through systematic visual inspection of the reconstructed meshes (Figure 8). The voting scheme successfully reconciled the conflicting predictions that arise when a single mesh face appears in multiple views, while the graph-diffusion step propagated label information into faces and vertices that were never directly observed—the geometric holes caused by occlusion. The procedure consistently eliminated speckle noise and filled occlusion-related gaps, producing watertight, manifold labeled surfaces suitable for the subsequent volumetric processing. Mesh integrity was verified quantitatively through bounding-box analysis and center-point verification, which confirmed that the global dimensions and the geometric origin were preserved during the transfer between processing environments, with no detectable displacement or scale distortion of the reconstructed geometry.

Beyond this qualitative and geometric verification, we quantified the accuracy of the back-projected labels against the original per-vertex ground truth on 60 scan-level held-out scans, using the auxiliary UNet++ trained on the remaining 240 scans (Section 2.4). On these unseen scans, the same model reaches a per-view mIoU of 62.04% (SD 15.09) and Dice of 65.24% (SD 15.81), whereas after probability-aggregation voting and graph-diffusion, the mesh-level mIoU rises to 87.36% (SD 12.91; median 92.74%) and Dice to 91.25% (SD 11.92)—a gain of +25.3 mIoU and +26.0 Dice points measured on the same scans with the same network (Table 5). The gain is systematic rather than average-driven: every one of the 60 held-out scans improves from the per-view to the mesh-level metric (range +7.8 to +46.9 mIoU points; median +20.7), with the largest gains on upper-jaw scans (52.5–90.1% mIoU). The few remaining low-scoring scans are those on which the per-view predictions were already weak (two scans below 50% mesh-level mIoU), indicating that aggregation consolidates good evidence rather than manufacturing it. Per-class results (Supplementary Table S2) show that the gain is broad across the dentition, while the third molars, represented by only five held-out scans, remain the weakest classes: teeth 18, 38 and 48 improve at the mesh level (43.3–46.7%, 60.8–73.7% and 32.1–43.0% IoU), whereas tooth 28 declines (30.6–24.4%), so no claim of third-molar recovery is made from this analysis. Pooled over all faces, mesh-level mIoU is 79.04% and Dice 87.31%, against pooled per-pixel values of 76.87% and 85.88% for the per-view predictions.

### 3.5. Occlusal-Plane Estimation and Splint Synthesis

Following automated occlusal-plane estimation, in which the SVD-derived normal vector and Rodrigues’ rotation aligned the dentition to the global reference frame (Supplementary Figure S3), the volumetric splint was synthesized and assessed for structural integrity. The normal-based displacement method produced a shell whose thickness is uniform by construction along each vertex normal; the nearest-surface wall thickness measured against the inner shell averaged 2.08 mm (SD 0.57 mm) against the 2.5 mm target, with the deviation concentrated at regions of high curvature such as cusp tips and interproximal embrasures (Section 2.7). The topological-dilation stage reliably produced the intended gingival extension of approximately 2.5 mm beyond the cervical line; the resulting geometry was closed and manifold across the majority of its connected shell components (Section 2.7), with a small, localized exception traced to a specific non-manifold cause. As illustrated in Supplementary Figure S4, the same pipeline generated both tooth-borne and gingiva-extended designs by modulating the number of dilation steps, demonstrating the clinical flexibility of the framework without any manual remodeling.

**Figure 9:**
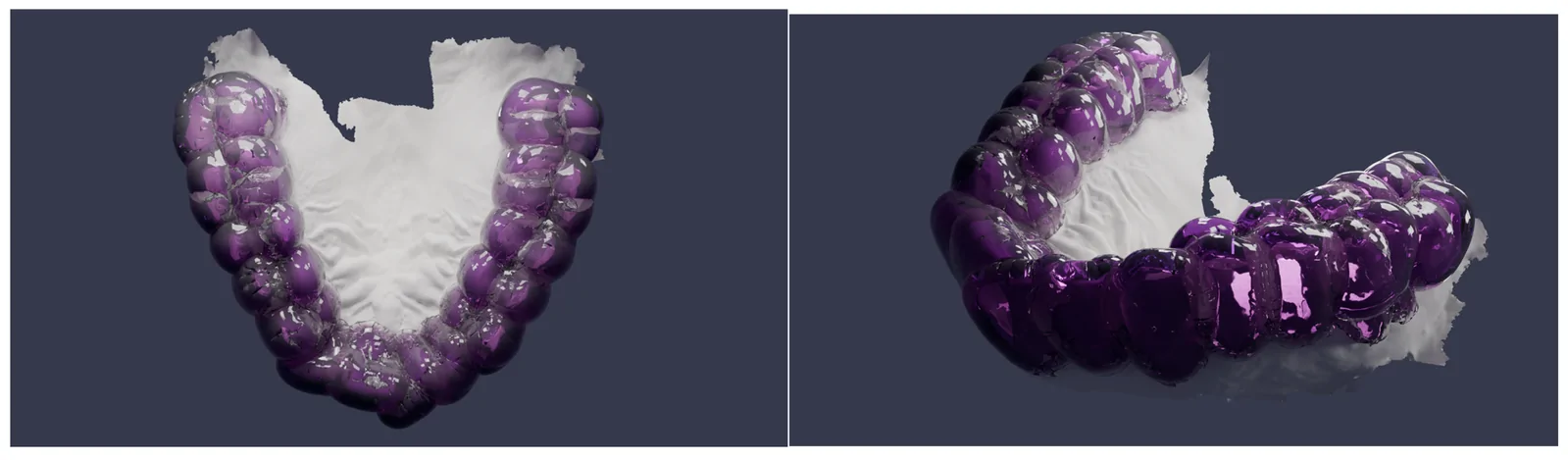
Physically based path-traced render of the synthesized splint (Blender Cycles), shown from multiple viewpoints.

The qualitative integrity of the final synthesis was further validated through high-fidelity, physically based path-traced rendering (Figure 9). A transparent thermoplastic-resin shader (index of refraction 1.49, high transmission weight) combined with a volume-absorption node allowed for direct visual inspection of the internal fit and the volumetric thickness between the splint and the underlying dentition. The renders confirm that the automated synthesis preserves the patient-specific occlusal topography and marginal boundaries and yields a manifold, 3D-printable geometry.

In the absence of clinician-annotated reference planes for the Teeth3DS+ dataset, the occlusal-plane estimator’s self-consistency was evaluated directly: the recovered plane normal was invariant to rigid rotation of the input mesh (0.000° mean deviation across 30 random orientations, as expected for an SVD-based estimator operating on centroid positions), and remained stable under simulated scanner noise, with only 0.16° mean angular deviation from the clean-mesh estimate at a 0.5 mm noise level representative of intraoral scanner precision. While this does not establish clinical accuracy against a ground-truth reference plane, it demonstrates that the estimate is not an artifact of mesh orientation or acquisition noise.

### 3.6. Computational Performance

**Figure 10:**
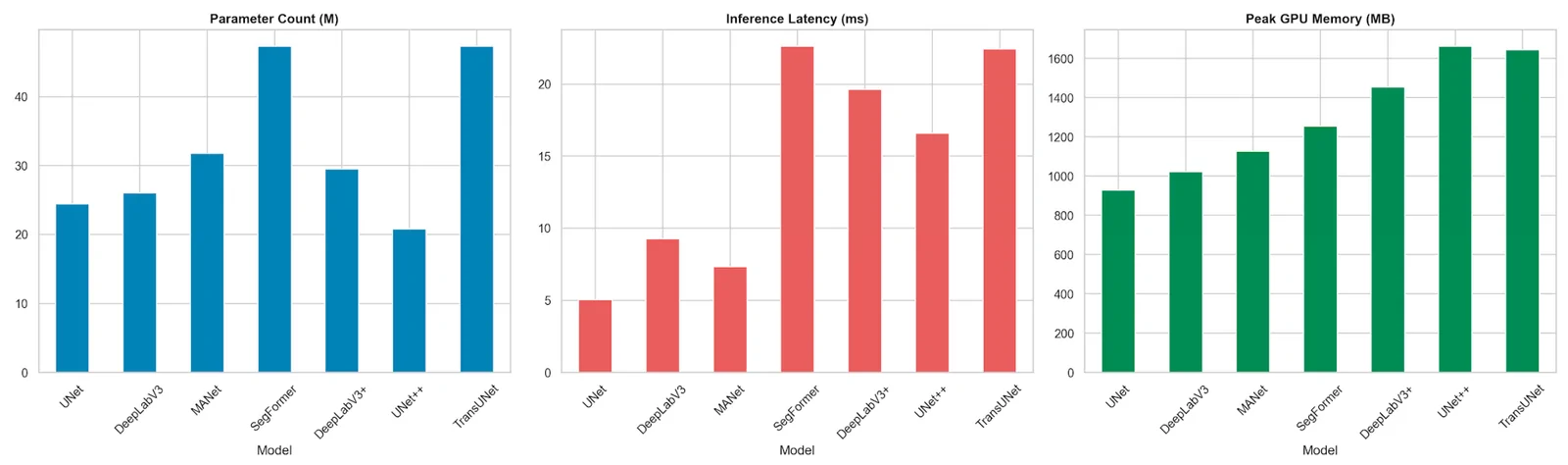
Computational cost of the seven architectures: parameter count, inference latency, and peak GPU memory.

To characterize the practical deployment cost of each architecture, we profiled parameter count, on-disk size, peak GPU memory, single-image inference latency, and throughput under identical conditions; the results are reported in Table 6 and Figure 10.

UNet++ achieves the highest segmentation accuracy while having the smallest parameter count (20.82 M) and the smallest on-disk size of the pool, at the cost of one of the two highest peak GPU memory footprints (1.65 GB, marginally below TransUNet’s 1.66 GB) due to its densely nested skip pathways. The lightweight UNet baseline is by far the fastest (174.1 FPS, 5.7 ms latency) but trails UNet++ by ≈3.3 mIoU points; SegFormer is the most expensive in latency (≈20.5 ms) without surpassing UNet++ in accuracy, whereas TransUNet, despite the largest parameter count of the pool (49.3 M), achieves substantially lower latency (7.6 ms) than SegFormer—comparable to the mid-weight CNN architectures—reflecting the efficiency of its convolutional decoder relative to SegFormer’s transformer-heavy design.

All experiments were conducted on a workstation equipped with a single NVIDIA RTX-class GPU (8 GB VRAM), an 8-core CPU, and 32 GB of system memory, using the PyTorch (v2.5.1) framework together with the PyTorch3D (v0.7.8) rendering library. Under this configuration, training each segmentation network for 10 epochs on the 300-model training set (9600 rendered views) required approximately 4 to 6 h. At inference, the complete pipeline processed a single intraoral scan in well under two minutes end-to-end: multi-view rendering of the 32 views together with network prediction accounted for the majority of the time (on the order of 30 to 60 s), while back-projection with graph diffusion, occlusal-plane estimation, and volumetric splint synthesis was completed within a comparable interval. This runtime is substantially shorter than the 20 to 40 min typically required for the manual CAD of an equivalent appliance, highlighting the throughput advantage of the automated workflow. The reported timings should be regarded as indicative, as they depend on mesh resolution and hardware configuration.

## 4. Discussion

Digital splint fabrication currently relies on the manual delineation of dental structures and on operator-dependent appliance design, which introduces variability into the diagnostic and treatment planning workflow and increases production time. The framework proposed in this study addresses this limitation by integrating fully automated diagnostic segmentation, occlusal-plane estimation, and splint synthesis directly from intraoral meshes, thereby streamlining the pathway from 3D diagnostic imaging to therapeutic intervention. To the best of our knowledge, this study is among the first frameworks to combine diagnostic tooth segmentation, automated occlusal-plane estimation, and volumetric splint synthesis within a single, end-to-end workflow.

### 4.1. Automated Tooth Segmentation in Context

Semantic segmentation of intraoral scans is an established research area with direct diagnostic implications, and several mesh- and point-cloud-based architectures define its current state of the art. MeshSegNet introduced multi-scale geometric feature learning directly on raw dental surfaces [11], TSegNet improved both efficiency and accuracy through a centroid-guided two-stage design [12], and two-stream graph convolutional networks exploited the distinct geometric meaning of coordinates and surface normals to refine inter-tooth boundaries [19]. More recent work, including two-stage mesh deep learning with iMeshSegNet [20] and graph-neural formulations such as TeethGNN [21], reports tooth-level accuracies in the high-ninety-percent range on clinical meshes. On the same Teeth3DS benchmark used here, the multi-stage TSegLab pipeline reports teeth-segmentation and identification rates above 97% [22]. In the present multi-view back-projection pipeline, the strongest configuration (UNet++) reaches a Mean Pixel Accuracy of 98.75%, a mean Intersection-over-Union of 71.76%, and a Dice coefficient of 75.20% on the rendered views, with the transformer- and attention-based architectures (TransUNet, DeepLabV3+, SegFormer) forming a statistically indistinguishable second tier. These figures are computed on the 2D-rendered views rather than on mesh cells. The quantity closest in kind to the surface-based metrics reported for 3D-native methods is the held-out mesh-level result of Section 3.4 (Table 5), obtained on 60 scan-level held-out scans that the evaluated network had never seen: mIoU 87.4% and Dice 91.3% after back-projection. Table 7 places this figure alongside the accuracy metrics reported in the original publications of the 3D-native methods introduced above. Because these methods differ in dataset (only TSegLab [22] uses the same Teeth3DS lineage), evaluation metric (DSC, mIoU, or identification rate) and train/test protocol, the values in Table 7 should be read as literature context rather than a head-to-head benchmark; within that caveat, they indicate that 3D-native architectures achieve higher segmentation accuracy than the multi-view back-projection approach: TSegLab reports segmentation and identification rates above 97%, MeshSegNet and TSegNet achieve DSC values in the 91–94% range, and TeethGNN reaches high-ninety-percent tooth-level accuracy, whereas our held-out mesh-level result stands at mIoU 87.4%/Dice 91.3%. The contribution of this work does not rest on surpassing these segmentation scores. Rather, it is twofold: architecturally, the pipeline relies solely on standard 2D encoder–decoder networks, avoids the memory cost and bespoke engineering required by dense 3D operators, and is more accessible for clinical deployment on commodity hardware; and in terms of pipeline scope, it is—to our knowledge—the only framework that does not terminate at the labeled surface but continues through automated occlusal-plane estimation and volumetric splint synthesis to deliver a 3D-printable appliance without manual CAD intervention.

### 4.2. From Segmentation to Appliance Generation

The principal contribution of this work is the transition from a labeled surface to a clinically usable appliance. The majority of published studies on intraoral segmentation terminate at the labeling stage and do not produce a printable device [11,12,19,20,21,22]. Conversely, studies that do generate occlusal splints generally depend on commercial CAD environments and operator interaction: fully digital splint workflows have been described using individualized mandibular-movement recordings [13] and through quantitative comparisons of CAD-CAM and analog devices [14], but these still require manual design steps performed by a clinician or technician. Even artificial-intelligence-assisted splint design has so far been benchmarked against a human technician rather than operating without manual delineation [15]. The framework presented here closes this gap by deriving the splint geometry algorithmically—through topological dilation for gingival extension and normal-based displacement for uniform thickness—from the segmented mesh, without manual point selection or template fitting.

### 4.3. Implications for the Diagnostic and Therapeutic Workflow

Three workflow-level benefits follow from this automation. First, laboratory and chairside time is reduced, because the steps that conventionally require a technician to outline structures and sculpt the appliance are replaced by a deterministic geometric pipeline; reduced chairside and adjustment time has been reported as a primary advantage of digital splint fabrication [13,14]. Second, operator dependency is lowered. The trueness of occlusal devices has been shown to vary with the operator and the design program used [15], and the proposed pipeline removes this source of variability by applying identical geometric rules to every case. Third, the workflow improves standardization and reproducibility. Error accumulation across the steps of the digital imaging chain is a recognized limitation, and the integration of artificial-intelligence-based steps has been proposed as the means to make the workflow simpler, more accurate, and more reproducible [7]; the expanding role of deep learning in dental image analysis supports this direction [6].

### 4.4. Clinical Relevance of the Design Parameters

The geometric parameters used in the synthesis stage were selected to match an established clinical device rather than chosen arbitrarily. The synthesized appliance corresponds to a full-coverage hard occlusal stabilization splint of the Michigan type, the design most commonly indicated for the management of bruxism and TMDs [3,5]; it is therefore distinct from a thin orthodontic retainer. A uniform shell thickness of approximately 2.5 mm was applied because full-coverage stabilization splints are conventionally fabricated with an occlusal thickness in the order of 2 mm, which provides adequate fracture resistance and allows even bilateral contacts to be established in centric relation [3]. The gingival extension of approximately 2.5 mm beyond the cervical line was applied to improve mechanical retention and seating of the appliance on the dentition. Both parameters are exposed as adjustable inputs in the pipeline, so the same framework can produce either a tooth-borne or a gingiva-extended design according to the clinical indication.

### 4.5. Diagnostic Value and Limitations of Per-Class Segmentation Performance

The per-class analysis reported in Section 3.3 has direct implications for the diagnostic reliability of the proposed framework. The near-ceiling IoU achieved on gingiva (0.97–0.98), background (0.99–1.00), and the anterior and mid-arch teeth (0.73–0.95) indicates that the pipeline reliably identifies and delineates the majority of clinically relevant structures—a prerequisite for automated dental charting, arch-form analysis, and the detection of missing or supernumerary teeth. Conversely, the comparatively lower performance on third molars (FDI 18, 28, 38, 48), where IoU ranges from 0.17 to 0.81 depending on the architecture and specific tooth, represents a diagnostically relevant limitation: third molars are frequently the subject of clinical diagnostic decisions (impaction assessment, extraction planning, pericoronitis evaluation), and their imprecise boundary delineation—rather than outright non-detection, given the moderate recall values reported in Section 3.3—would still warrant clinician verification in any deployed system. The finding that this gap narrowed substantially once a validated learning rate was adopted (Section 2.3)—rather than through architecture choice or loss-function tuning alone (Section 4.6)—suggests that training-protocol selection is at least as consequential for diagnostic reliability on rare classes as architecture selection itself. These results underscore that while the framework provides a robust foundation for computer-aided diagnostic assessment of the dental arch, a human-in-the-loop verification step remains clinically advisable—particularly for the posterior dentition—before the diagnostic output is used to guide treatment planning or appliance fabrication.

### 4.6. Limitations and Future Work

Several limitations should be acknowledged. The framework was developed and evaluated on a single public benchmark (Teeth3DS+), which predominantly comprises scans of structurally intact, non-pathological dentitions; no cases involving missing teeth, dental implants, crowns, bridges, severe occlusal wear, or dental crowding were represented in the training or test data, and the pipeline’s performance on such cases remains unevaluated. This limitation compounds the class imbalance already present in the healthy-dentition data used here—background and gingiva account for 87.5% of labeled pixels, and the four third molars collectively account for only 0.155% (Table S1)—since pathological presentations would be expected to further skew, or altogether depart from, this distribution. Generalization to intraoral scans acquired with different scanners or acquisition protocols has also not been tested. The held-out mesh-level evaluation (Section 3.4) rests on a 60-scan split drawn from the training/validation pool and on an auxiliary network trained on the remaining 240 scans, because the original meshes of the 99-scan test set were not retained after rendering; its third-molar estimates in particular rely on only five held-out scans and should be read with correspondingly wide uncertainty.

A further limitation concerns the training protocol. The segmentation models were trained for ten epochs, and the per-class analysis indicates that the rare distal molars and the narrow inter-tooth boundaries remain the principal sources of residual diagnostic error even for the best model (per-image mIoU 71.76%, UNet++). We tested one candidate mitigation directly: an ablation comparing the deployed Dice + Focal loss against plain cross-entropy on UNet, at the originally planned learning rate, found cross-entropy achieving substantially higher third-molar IoU (mean 0.37 vs. 0.07 across teeth 18/28/38/48), indicating that the imbalance-aware loss alone was not the most effective lever for this architecture. The subsequent adoption of a validated learning rate (Section 2.3) had a considerably larger effect on third-molar recovery than this loss-function change, suggesting that optimization dynamics play at least as large a role as loss design or architecture choice in rare-class performance. Boundary-aware losses and further per-architecture hyperparameter tuning remain plausible directions for narrowing the residual gap further. The current synthesis establishes the splint geometry but does not yet incorporate dynamic occlusion, condylar guidance, or virtual-articulator-based occlusal refinement, all of which are clinically relevant for a definitive stabilization splint [13]. Extending the splint-synthesis stage with undercut block-out, individual occlusal-contact modeling, and jaw-movement-based refinement—building on the geometric quality metrics established in Section 2.7—is a natural next step toward a clinically deployable appliance. Furthermore, the automated occlusal-plane estimation, while providing a quantitative and reproducible diagnostic measurement, is based on a planar approximation that may not adequately represent the curve of Spee or account for significant malocclusions; its diagnostic accuracy against clinician-determined reference planes has not yet been formally evaluated. A self-consistency check confirms the estimator is stable under rotation and realistic scan noise, but this does not substitute for validation against a clinical reference plane, which remains necessary before the measurement can be used diagnostically. Finally, validation in this study is geometric and qualitative; in vivo assessment of diagnostic accuracy, splint fit, occlusal contact distribution, and material wear on printed appliances has not been performed. Accordingly, the present study should be interpreted as a proof-of-concept demonstrating the feasibility of an automated diagnostic-to-intervention digital workflow rather than a clinically validated diagnostic tool or manufacturing protocol. Future work will extend the pipeline toward missing-tooth detection, occlusal-plane deviation analysis as a diagnostic marker for TMDs, automated implant-position analysis, and prospective clinical validation of both the diagnostic outputs and the printed devices.

Unlike previous studies that focused on isolated stages such as segmentation or CAD-based appliance design, the present framework demonstrates that AI-driven diagnostic understanding of intraoral meshes—encompassing tooth identification, spatial classification, and occlusal-plane assessment—can be directly translated into a manufacturable therapeutic appliance without user intervention. This establishes a foundation for future computer-aided diagnostic and therapeutic workflows in digital dentistry, including automated dental charting, missing-tooth detection, implant planning, prosthetic design, and personalized oral appliance fabrication.

## 5. Conclusions

This study presents a fully automated pipeline that converts raw intraoral 3D meshes into patient-specific Michigan-type stabilization splints through deep learning-based segmentation, multi-view back-projection, occlusal-plane estimation, and volumetric splint synthesis—without manual CAD modeling. Among seven benchmarked architectures, UNet++ achieved the strongest performance (MPA 98.75%, mIoU 71.76%, Dice 75.20%), with statistically significant superiority over all competitors, while probability-aggregation voting and graph diffusion raised mesh-level mIoU by 25 points over per-view predictions on held-out scans. Per-class analysis revealed near-ceiling accuracy on well-represented teeth and residual error concentrated on third molars, highlighting the sensitivity of rare-class segmentation to training hyperparameters and the continued need for clinician verification on the posterior dentition. The framework demonstrates that AI-driven diagnostic understanding of intraoral geometry can extend beyond surface labeling into a complete diagnostic-to-intervention workflow, with future work targeting missing-tooth detection, occlusal-plane deviation analysis, and prospective clinical validation of both segmentation outputs and fabricated appliances.

## Figures and Tables

**Table 1:** Dataset composition and multi-view rendering configuration.

Property	Value
Dataset	Teeth3DS+ (primary partition)
Total 3D scans	399
Training/validation scans	300
Test scans	99
Views rendered per scan	32
Total training/validation views	9600
Total test/evaluation views	3168
Files per scan	.obj geometry + .json metadata
Number of classes	34 (background, gingiva, 32 FDI teeth)
Input modalities per view	RGB render, surface normal map, FDI label map

**Table 2:** Training hyperparameters.

Hyperparameter	Value
Optimizer	AdamW
Epochs	10
Loss Function	Dice Loss + Focal Loss (equal weighting, 0.5/0.5)
Learning Rate	2 × 10^−4^
Batch Size	4

**Table 3:** Comparative segmentation performance on the held-out test set (mean ± standard deviation over 3168 rendered views). Best value per column in bold.

Model	Mean Pixel Accuracy (%)	mIoU (%)	Dice (%)
UNet	98.37 ± 1.52	68.49 ± 15.84	72.68 ± 16.38
DeepLabV3	98.12 ± 1.38	68.27 ± 14.77	73.30 ± 15.50
MANet	98.24 ± 1.65	66.71 ± 16.55	70.93 ± 17.08
SegFormer	98.38 ± 1.55	70.12 ± 15.29	74.36 ± 15.89
DeepLabV3+	98.37 ± 1.38	70.17 ± 15.35	74.44 ± 15.94
UNet++	**98.75 ± 1.41**	**71.76 ± 15.92**	**75.20 ± 16.31**
TransUNet	98.61 ± 1.45	70.29 ± 15.86	74.04 ± 16.28

**Table 4:** Statistical significance of selected pairwise mIoU differences (paired *t*-test and Wilcoxon signed-rank test) at the image level (n = 3168 views): UNet++ versus every other architecture, together with the three mid-cluster comparisons for which the two tests disagreed. The complete 21-comparison matrix at the scan level is provided in Supplementary Table S4. Δ = mean difference (A − B).

Model A	Model B	ΔmIoU	t *p*-Value	Wilcoxon *p*	Significance
UNet++	UNet	+3.26	<0.001	<0.001	✓
UNet++	DeepLabV3	+3.48	<0.001	<0.001	✓
UNet++	MANet	+5.04	<0.001	<0.001	✓
UNet++	SegFormer	+1.63	<0.001	<0.001	✓
UNet++	DeepLabV3+	+1.59	<0.001	<0.001	✓
UNet++	TransUNet	+1.46	<0.001	<0.001	✓
SegFormer	TransUNet	−0.17	0.146	<0.001	✗ (*t*-test)
SegFormer	DeepLabV3+	−0.04	0.666	<0.001	✗ (*t*-test)
DeepLabV3+	TransUNet	−0.13	0.276	<0.001	✗ (*t*-test)

**Table 5:** Held-out evaluation of the back-projection stage: per-view 2D versus mesh-level metrics on 60 scan-level held-out scans, using the auxiliary UNet++ trained on the remaining 240 scans (Section 2.4). Mean ± SD over scans (per-view metrics are first averaged over the 32 views of each scan); pooled values aggregate all pixels or faces.

Metric	Per-View 2D	Mesh-Level	Δ	Pooled 2D	Pooled Mesh
Accuracy (%)	98.31 ± 1.53	93.05 ± 9.50	n/a †	-	-
mIoU (%)	62.04 ± 15.09	87.36 ± 12.91	+25.32	76.87	79.04
mDice (%)	65.24 ± 15.81	91.25 ± 11.92	+26.01	85.88	87.31

† Per-view accuracy is dominated by background pixels (≈60% of every view), which have no counterpart on the mesh surface; the two accuracy figures are therefore not comparable.

**Table 6:** Computational cost of the evaluated architectures (single NVIDIA RTX-class GPU, 8 GB VRAM).

Model	Params (M)	Size (MB)	Peak GPU (MB)	Latency (ms)	FPS	Eval (ms/img)
UNet	24.44	93.3	929	5.74	174.1	9.19
DeepLabV3	26.02	99.3	1023	9.14	109.5	12.97
MANet	31.79	121.3	1127	7.17	139.4	12.59
SegFormer	44.61	170.2	1296	20.52	48.7	34.34
DeepLabV3+	29.50	113.2	1443	18.49	54.1	31.20
UNet++	20.82	79.9	1651	19.86	50.4	31.10
TransUNet	49.25	188.1	1661	7.56	132.2	13.79

**Table 7:** Reported segmentation accuracy of 3D-native methods discussed in Section 4.1, alongside the present pipeline’s mesh-level result.

Method	Evaluation Dataset	Reported Metric(s)	Value (%)
MeshSegNet [11]	Private clinical dataset (100 patients, as re-evaluated in [20])	DSC	86.2
TSegNet [12]	Private clinical dataset (2000 scans, 400 test)	DSC (surface)/F1 (identification)	98.6/94.2
TeethGNN [21]	3DTeethSeg’22 benchmark	mIoU	83.9
TSegLab [22,23]	Teeth3DS (1800 scans)	Segmentation accuracy/Identification rate	98.2/97.6
Ours-mesh-level after back-projection (UNet++)	Teeth3DS+, 60 scan-level held-out scans (Section 2.4)	mIoU/Dice	87.4/91.3

## Data Availability

The original data used in this study are openly available in the Teeth3DS+ benchmark dataset [23]. Additional data generated during the study are available from the corresponding author upon reasonable request.

## Supplementary Materials

The following supporting information can be downloaded at https://www.mdpi.com/article/10.3390/diagnostics16183039/s1. Supplementary Table S1. Per-class ground-truth pixel distribution across the training/validation views (9600 rendered views from 300 scans) and the held-out test set (3168 rendered views from 99 scans). Percentages are computed within each split independently; the ‘Total’ columns pool both splits. Supplementary Table S2. Per-class per-view 2D versus mesh-level IoU and Dice on the 60 scan-level held-out scans, both computed with the auxiliary UNet++ of Section 2.4 (pooled over all views/faces). Background is omitted because it has no faces on the mesh. Supplementary Table S3. Validation-only hyperparameter sweep on UNet++ (abbreviated protocol: 1920 training/480 validation views). Supplementary Table S4. Scan-level significance testing of all 21 pairwise mIoU differences (per-image scores averaged within each scan, n = 99; paired *t*-test and Wilcoxon signed-rank test). Δ = mean difference (A − B) in percentage points. Supplementary Figure S1. Example RGB render, surface normal map, and FDI label map for a single rendered view. Supplementary Figure S2. The back-projected and graph-diffused labeled mesh, shown from three viewpoints (occlusal and two oblique angles), demonstrating a continuous, denoised labeled surface. Supplementary Figure S3. Occlusal-plane estimation: tooth centroids (red) and the SVD-fitted plane (blue), before alignment (left) and after Rodrigues-rotation alignment to the global coordinate frame (right). Supplementary Figure S4. Tooth-borne (Version A, 0 mm gingival extension) and gingiva-extended (Version B, 2.5 mm gingival extension) splint designs generated by the same pipeline through modulation of the topological-dilation step count (Section 2.7).

## Abbreviations

The following abbreviations are used in this manuscript:

ASPP	Atrous Spatial Pyramid Pooling
CAD	Computer-Aided Design
CAM	Computer-Aided Manufacturing
CNN	Convolutional Neural Network
DSC	Dice Similarity Coefficient
FDI	Fédération Dentaire Internationale (tooth numbering)
mIoU	Mean Intersection-over-Union
MPA	Mean Pixel Accuracy
SVD	Singular Value Decomposition
TMD	Temporomandibular Disorder
