# Supplementary material for: Automated 3D Tooth Segmentation and Dental Splint Synthesis from Intraoral Scans: A Deep Learning Pipeline for Computer-Aided Digital Dentistry
Source: Diagnostics (Basel). 2026 Sep 19;16(18):3039. doi: 10.3390/diagnostics16183039 (PMC13605753; doi:10.3390/diagnostics16183039)
Supplement: Supplementary file 1 [file diagnostics-16-03039-s001.zip › diagnostics-4528408-supplementary.pdf]

diagnostics

MDPI

Supplementary Material

Supplementary Table S1. Per-class ground-truth pixel distribution across the training/validation views (9600 rendered views from 300 scans) and the held-out test set (3168 rendered views from 99 scans). Percentages are computed within each split independently; the 'Total' columns pool both splits.

class_id	Class	Train/Val pixels	Train/Val %	Test pixels	Test %	Total pixels	Total %
0	Background	593,389,414	60.363	193,496,131	59.647	786,885,545	60.185
1	Gingiva	264,658,637	26.923	92,522,909	28.512	357,181,546	27.319
2	Tooth 18	385,797	0.039	69,541	0.021	455,338	0.035
3	Tooth 17	5,232,009	0.532	719,618	0.222	5,951,627	0.455
4	Tooth 16	8,008,945	0.815	1,205,339	0.372	9,214,284	0.705
5	Tooth 15	4,264,986	0.434	613,799	0.189	4,878,785	0.373
6	Tooth 14	3,984,449	0.405	563,303	0.174	4,547,752	0.348
7	Tooth 13	3,673,629	0.374	480,393	0.148	4,154,022	0.318
8	Tooth 12	3,081,804	0.314	468,876	0.145	3,550,680	0.272
9	Tooth 11	4,804,663	0.489	691,829	0.213	5,496,492	0.420
10	Tooth 21	4,895,476	0.498	705,087	0.217	5,600,563	0.428
11	Tooth 22	3,256,184	0.331	485,763	0.150	3,741,947	0.286
12	Tooth 23	3,832,185	0.390	562,353	0.173	4,394,538	0.336
13	Tooth 24	4,264,347	0.434	619,129	0.191	4,883,476	0.374
14	Tooth 25	4,316,476	0.439	661,242	0.204	4,977,718	0.381
15	Tooth 26	8,078,610	0.822	1,249,501	0.385	9,328,111	0.714
16	Tooth 27	5,324,870	0.542	654,832	0.202	5,979,702	0.457
17	Tooth 28	370,152	0.038	37,089	0.011	407,241	0.031
18	Tooth 31	2,642,396	0.269	1,314,265	0.405	3,956,661	0.303
19	Tooth 32	2,769,017	0.282	1,378,882	0.425	4,147,899	0.317
20	Tooth 33	3,285,369	0.334	1,623,934	0.501	4,909,303	0.376
21	Tooth 34	3,249,600	0.331	1,622,113	0.500	4,871,713	0.373
22	Tooth 35	3,641,509	0.370	1,803,884	0.556	5,445,393	0.417
23	Tooth 36	6,931,502	0.705	3,496,777	1.078	10,428,279	0.798
24	Tooth 37	5,107,724	0.520	2,615,604	0.806	7,723,328	0.591
25	Tooth 38	254,207	0.026	172,739	0.053	426,946	0.033
26	Tooth 48	515,278	0.052	223,696	0.069	738,974	0.057
27	Tooth 47	5,516,371	0.561	2,852,456	0.879	8,368,824	0.640
28	Tooth 46	7,260,413	0.739	3,678,394	1.134	10,938,807	0.837
29	Tooth 45	3,902,382	0.397	1,867,434	0.576	5,769,816	0.441
30	Tooth 44	3,561,175	0.362	1,701,722	0.525	5,262,897	0.403
31	Tooth 43	3,416,489	0.348	1,665,200	0.513	5,081,689	0.389
32	Tooth 42	2,739,438	0.279	1,356,985	0.418	4,096,423	0.313
33	Tooth 41	2,424,497	0.247	1,222,384	0.377	3,646,881	0.279

Supplementary Table S2. Per-class per-view 2D versus mesh-level IoU and Dice on the 60 scan-level held-out scans, both computed with the auxiliary UNet++ of Section 2.4 (pooled over all views / faces). Background is omitted because it has no faces on the mesh.

Class	2D IoU (%)	Mesh IoU (%)	Δ	2D Dice (%)	Mesh Dice (%)	Mesh Support (faces)
Gingiva	98.05	95.73	-2.32	99.02	97.82	6,563,026
Tooth 18	43.26	46.65	+3.39	60.40	63.62	16,846
Tooth 17	75.25	80.25	+5.00	85.88	89.05	191,827
Tooth 16	82.66	85.95	+3.30	90.50	92.45	443,629
Tooth 15	78.53	81.98	+3.45	87.97	90.10	272,872
Tooth 14	86.84	88.75	+1.90	92.96	94.04	259,924
Tooth 13	89.01	91.30	+2.29	94.19	95.45	196,408
Tooth 12	86.75	89.83	+3.07	92.91	94.64	187,616
Tooth 11	92.65	93.53	+0.88	96.18	96.66	285,206
Tooth 21	92.57	93.71	+1.14	96.14	96.75	287,996
Tooth 22	87.59	90.72	+3.13	93.38	95.13	192,742
Tooth 23	88.48	91.01	+2.53	93.89	95.29	206,336
Tooth 24	85.71	88.84	+3.13	92.30	94.09	255,482
Tooth 25	76.72	82.83	+6.11	86.83	90.61	269,698
Tooth 26	81.38	85.57	+4.19	89.73	92.22	424,328
Tooth 27	74.45	76.96	+2.51	85.36	86.98	242,661
Tooth 28	30.60	24.36	-6.24	46.87	39.18	28,765
Tooth 31	69.01	75.43	+6.42	81.66	85.99	184,102
Tooth 32	71.65	74.57	+2.93	83.48	85.44	193,298
Tooth 33	78.62	78.08	-0.55	88.03	87.69	195,893
Tooth 34	77.99	78.29	+0.30	87.64	87.82	223,363
Tooth 35	65.59	65.99	+0.40	79.22	79.51	282,722
Tooth 36	75.44	73.88	-1.56	86.00	84.98	430,678
Tooth 37	78.13	78.62	+0.49	87.72	88.03	294,453
Tooth 38	60.75	73.66	+12.91	75.59	84.83	15,841
Tooth 48	32.05	42.96	+10.91	48.55	60.10	18,003
Tooth 47	70.71	75.40	+4.70	82.84	85.98	274,914
Tooth 46	73.14	76.30	+3.16	84.49	86.56	449,312
Tooth 45	74.29	77.03	+2.74	85.25	87.03	268,438
Tooth 44	86.00	90.66	+4.67	92.47	95.10	233,466
Tooth 43	86.13	89.25	+3.12	92.55	94.32	205,952
Tooth 42	83.83	88.22	+4.39	91.20	93.74	188,874
Tooth 41	79.79	82.03	+2.24	88.76	90.13	178,404

Supplementary Table S3. Validation-only hyperparameter sweep on UNet++ (abbreviated protocol: 1920 training / 480 validation views).

Configuration	Learning Rate	Batch Size	Epochs	Final Validation mIoU
lr5e-5_bs4	5×10^{-5}	4	3	0.055
lr1e-4_bs4	1×10^{-4}	4	3	0.096
lr2e-4_bs4	2×10^{-4}	4	3	**0.140**
lr1e-4_bs2	1×10^{-4}	2	3	0.125
lr1e-4_bs8	1×10^{-4}	8	3	0.058
lr1e-4_bs4 (extended)	1×10^{-4}	4	8	0.409
lr2e-4_bs4 (extended)	2×10^{-4}	4	8	**0.685**

Supplementary Table S4. Scan-level significance testing of all 21 pairwise mIoU differences (per-image scores averaged within each scan, n = 99; paired t-test and Wilcoxon signed-rank test). Δ = mean difference (A − B) in percentage points.

Model A	Model B	ΔmIoU	t p-value	Wilcoxon p	Significance (t-test)
UNet	DeepLabV3	+0.22	0.186	0.054	✗
UNet	MANet	+1.78	<0.001	<0.001	✓
UNet	SegFormer	-1.63	<0.001	<0.001	✓
UNet	DeepLabV3+	-1.67	<0.001	<0.001	✓
UNet	UNet++	-3.26	<0.001	<0.001	✓
UNet	TransUNet	-1.80	<0.001	<0.001	✓
DeepLabV3	MANet	+1.56	<0.001	<0.001	✓
DeepLabV3	SegFormer	-1.85	<0.001	<0.001	✓
DeepLabV3	DeepLabV3+	-1.89	<0.001	<0.001	✓
DeepLabV3	UNet++	-3.48	<0.001	<0.001	✓
DeepLabV3	TransUNet	-2.02	<0.001	<0.001	✓
MANet	SegFormer	-3.41	<0.001	<0.001	✓
MANet	DeepLabV3+	-3.45	<0.001	<0.001	✓
MANet	UNet++	-5.04	<0.001	<0.001	✓
MANet	TransUNet	-3.58	<0.001	<0.001	✓
SegFormer	DeepLabV3+	-0.04	0.794	0.092	✗
SegFormer	UNet++	-1.63	<0.001	<0.001	✓
SegFormer	TransUNet	-0.17	0.493	0.001	✗
DeepLabV3+	UNet++	-1.59	<0.001	<0.001	✓
DeepLabV3+	TransUNet	-0.13	0.691	<0.001	✗
UNet++	TransUNet	+1.46	<0.001	<0.001	✓

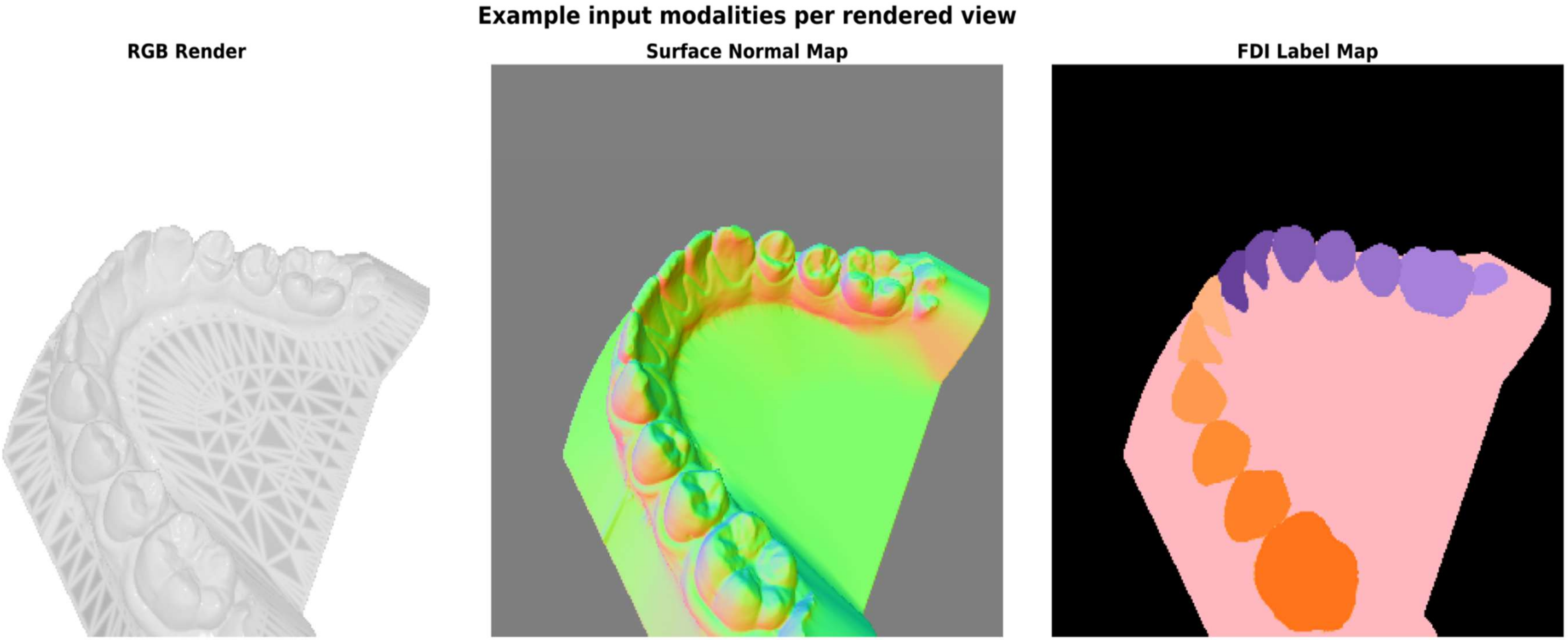

Supplementary Figure S1. Example RGB render, surface normal map, and FDI label map for a single rendered view.

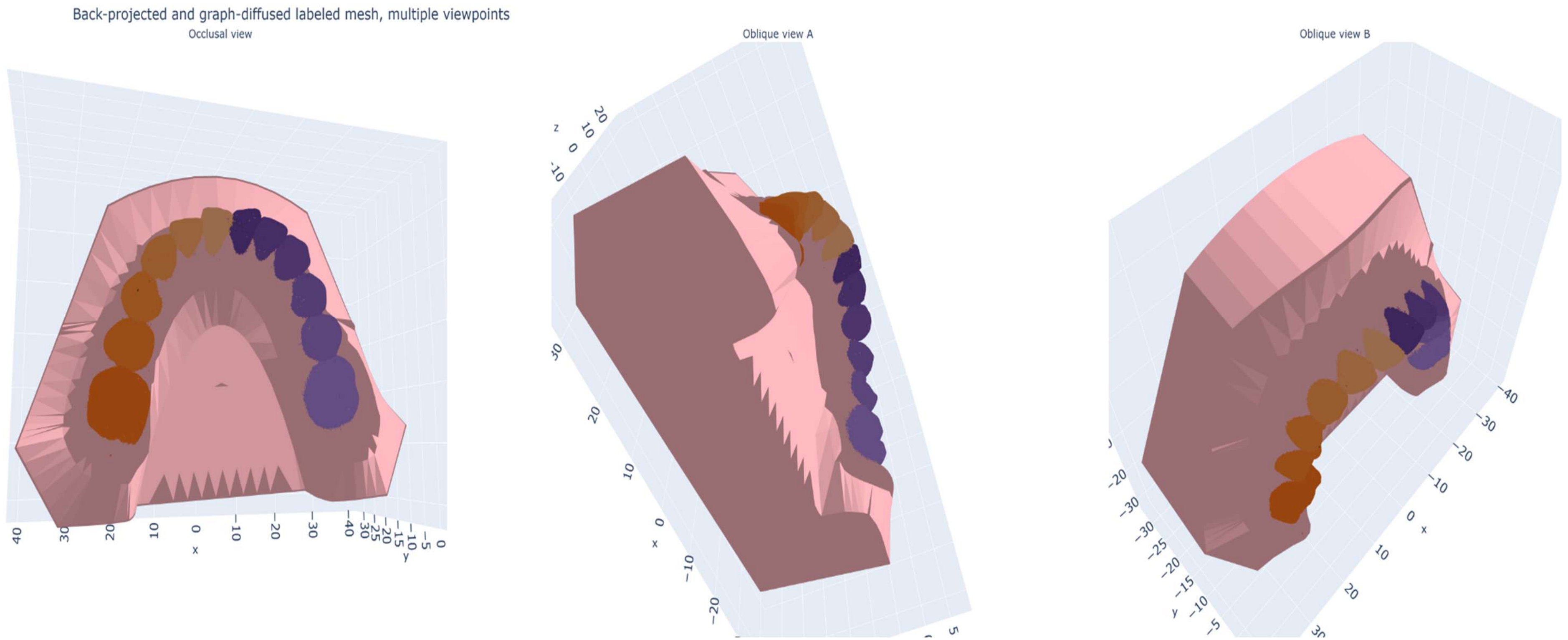

Supplementary Figure S2. The back-projected and graph-diffused labeled mesh, shown from three viewpoints (occlusal and two oblique angles), demonstrating a continuous, denoised labeled surface.

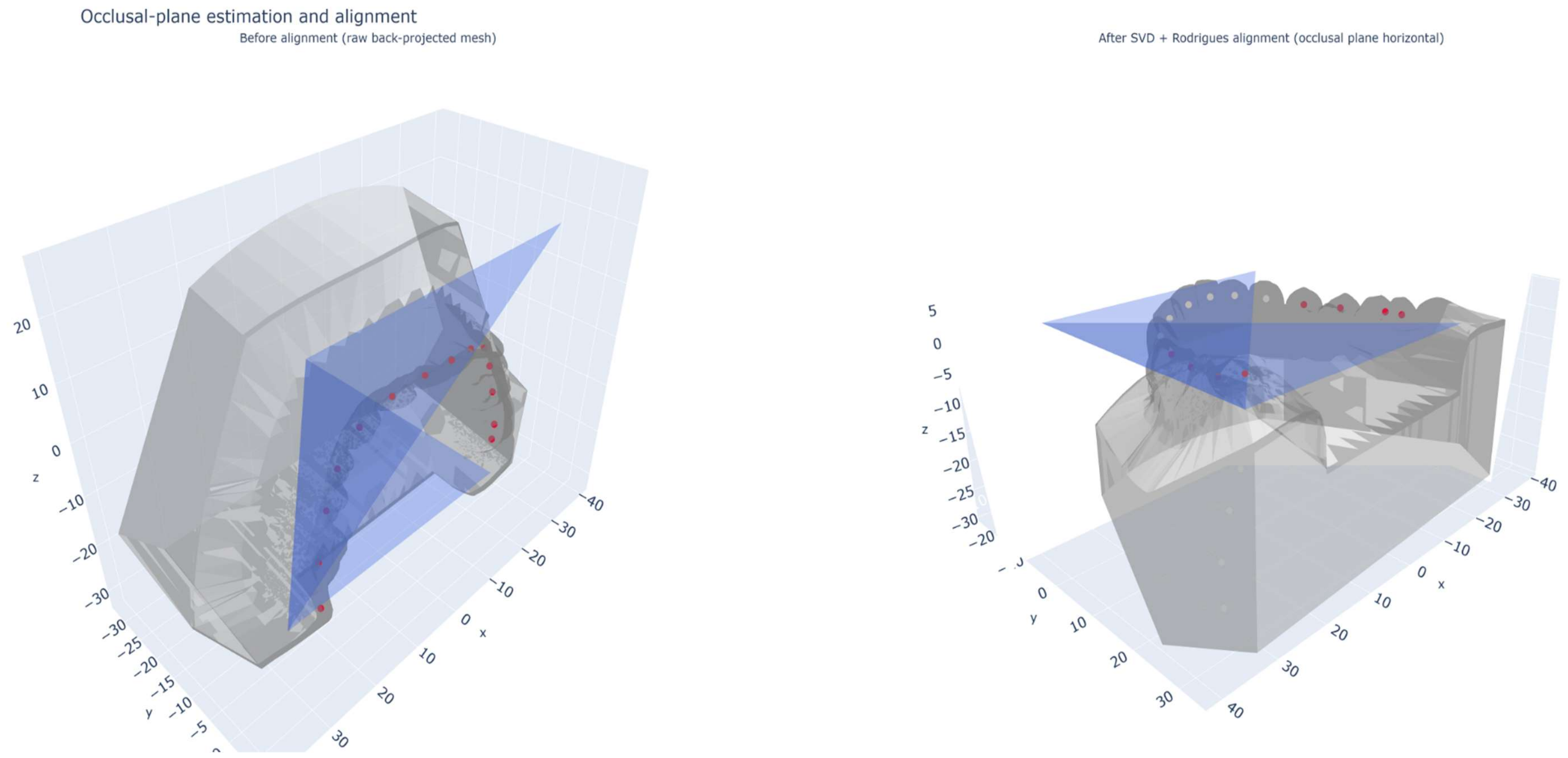

Supplementary Figure S3. Occlusal-plane estimation: tooth centroids (red) and the SVD-fitted plane (blue), before alignment (left) and after Rodrigues-rotation alignment to the global coordinate frame (right).

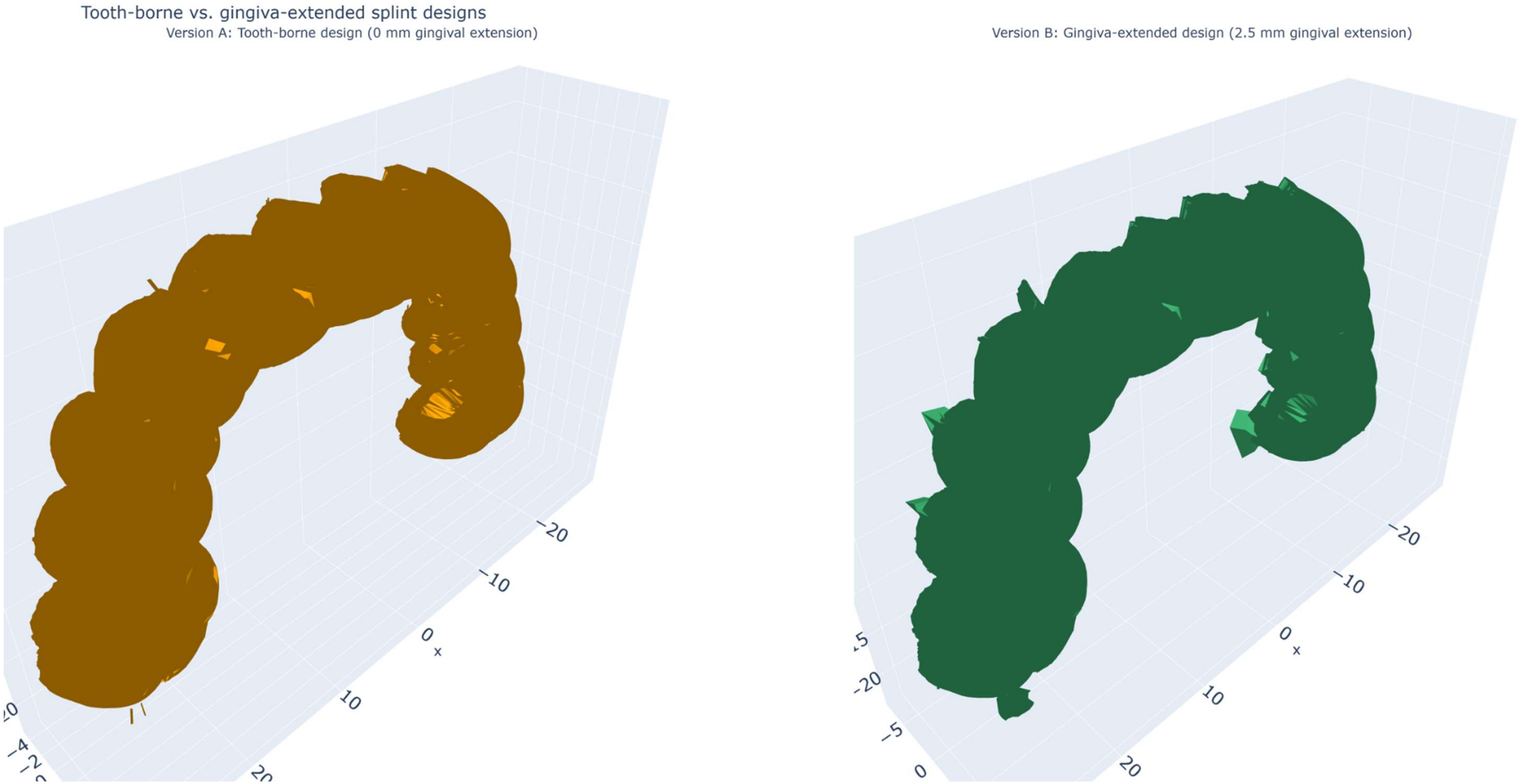

Supplementary Figure S4. Tooth-borne (Version A, 0 mm gingival extension) and gingiva-extended (Version B, 2.5 mm gingival extension) splint designs generated by the same pipeline through modulation of the topological-dilation step count (Section 2.7).
